# Widespread Emissions of Polychlorinated Biphenyls from Building Materials in Vermont Schools

**DOI:** 10.1021/acs.est.5c10939

**Published:** 2026-02-03

**Authors:** Jason B. X. Hua, Rachel F. Marek, Michael P. Jones, Trevor D. Erb, Sarah C. Owen, Keri C. Hornbuckle

**Affiliations:** † Department of Civil and Environmental Engineering, IIHR-Hydroscience and Engineering, The University of Iowa, Iowa City, Iowa 52242, United States; ‡ Department of Biostatistics, The University of Iowa, Iowa City, Iowa 52242, United States; § Division of Environmental Health, Vermont Department of Health, Waterbury 05671, United States

**Keywords:** PUF−PES, semivolatile organic compounds, persistent organic pollutants, PCBs, Aroclors, legacy compounds, building materials, school air

## Abstract

In collaboration with Vermont state and school officials, we conducted a research study to measure emissions of polychlorinated biphenyls (PCBs) from room surfaces in Vermont schools. Our study, the largest of its kind, investigated the sources of airborne PCBs in indoor school environments. Using simultaneous deployment of air samplers and emission samplers, we measured airborne PCBs in 16 schools and 98 school rooms constructed prior to 1980. There was a wide range in PCB air concentrations (1.7–5700 ng m^–3^, *n* = 159) and surface emissions (33–830,000 ng m^–2^ d^–1^, *n* = 182) across different schools as well as between rooms in the same school. We found that emissions of PCB congeners from walls, floors, ceiling and wall expansion joint caulking, and spray insulation explain the airborne PCB congener concentrations in many rooms. Our emission samplers identified three distinct types of building materials with emissions exceeding 30,000 ng m^–2^ d^–1^ including expansion joint sealant (up to 480,000 ng m^–2^ d^–1^), glass block windows (up to 30,000 ng m^–2^ d^–1^), and fireproof coating on steel columns (up to 830,000 ng m^–2^ d^–1^). Consequently, school staff have an estimated excess lifetime cancer risk from both dioxin-like and nondioxin-like PCBs that ranges from 1.3 × 10^–8^ to 1.7 × 10^–4^ for central tendency exposure, and 2.8 × 10^–8^ to 3.8 × 10^–4^ for reasonable maximum exposure (State of Vermont’s target cancer risk = 1 × 10^–6^). Although production has been banned for decades, our study illustrates that PCBs continue to pose an exposure risk to occupants due to their long history of use in building materials. Our findings underscore the risks associated with the historic presence of PCB-containing building materials, offering critical insights for community efforts aimed at reducing exposure among children and school staff in thousands of schools across the country.

## Graphical abstract

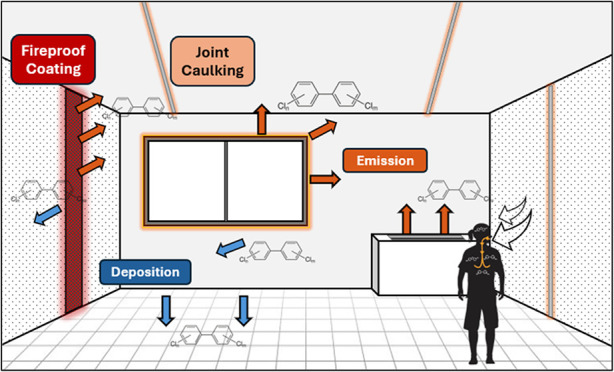

## Introduction

Polychlorinated biphenyls (PCBs) were identified as possible carcinogens in the middle part of the last century at a time when commercial use of PCB mixtures, called Aroclors in the United States (US), was widespread. While they were banned from production in the US in 1979, there is no timetable for removal or destruction of existing stock. Although the US is not a signatory, the Stockholm Convention banned PCBs from use and production and states a goal of elimination of PCB use in equipment by 2025 and environmentally sound management by 2028.^1^ Efforts in the US to control human exposure to PCBs have focused on reducing PCB concentrations in fish and contact with contaminated sediments, solid waste, and surface waters.^2–4^ Improvements in analytical methods and instrumentation in the 1980s demonstrated that PCBs are released from surfaces, both indoors and outdoors, as gases at ambient temperatures.^5–7^ However, regulation of airborne PCB concentrations indoors, in any US public building, has not been a consideration until recently.

It is now clear that Aroclors used in school construction continue to emit PCBs many decades after their initial installation. Caulking, sealants, and light ballasts were all known to contain Aroclor PCBs,^8,9^ but the full history of PCB addition to building materials is not known: PCBs were added during manufacturing of materials and/or added to the materials as the building was constructed. It is also evident that PCBs in building materials diffuse through solid materials and redeposit from air to surfaces, creating secondary and tertiary sources of emissions. Furthermore, non-Aroclor PCBs are an inadvertent byproduct of some chemical manufacturing processes still in use today for consumer products, such as paint pigments and silicone rubber. As a result, there are many potential sources of PCB emissions in school rooms and other buildings.^10–15^ PCB emissions from these Aroclor- and non-Aroclor containing materials in schools are likely to pose a significant risk of airborne exposure to occupants, including children, pregnant people, and long-time staff members.^16–29^ However, airborne concentrations of PCBs and their sources have not been widely assessed in US schools. Given the potential risk to human health, there is a critical need to identify sources of PCB contamination and reduce their levels in schools and public buildings.

In 2021, Vermont became the first state to directly regulate the release of PCBs from building materials into the air in schools and set grade-specific school action levels (SAL): 30 ng m^–3^ for pre-kindergarten (up to age 5), 60 ng m^–3^ for kindergarten to sixth grade (ages 5 to 12), and 100 ng m^–3^ for seventh grade to adults (age 13 and older).^30,31^ In addition, immediate action levels (IAL) were set as three times the SAL for each age group to advise schools on restricting access to contaminated rooms and accelerate corrective actions.^32^ Vermont also launched a first-in-the-nation air sampling program to identify any school rooms with airborne PCB levels above the SALs. The state prioritized measuring airborne PCBs in schools built in or before 1980 and has publicly released their findings for airborne PCB levels in over 100 schools and more than 4800 air samples.^31^ Although classrooms were the primary focus for sampling, measurements were also collected in gymnasiums, auditoriums, offices, kitchens, boiler rooms, utility rooms, and other ancillary spaces such as storage closets and server rooms. Most of these samples were collected using the EPA method TO-10A^33^ (Figure 1A), which provides only the total PCB air concentration as the sum of all reportable Aroclors and not individual congeners. However, air sampling alone does not identify sources of PCBs in classrooms. Direct emission measurements from suspected sources are needed to nondestructively determine what materials must be removed or remediated, yet there are no standard methods for direct measurement of emissions from individual surfaces.

**Figure 1:**
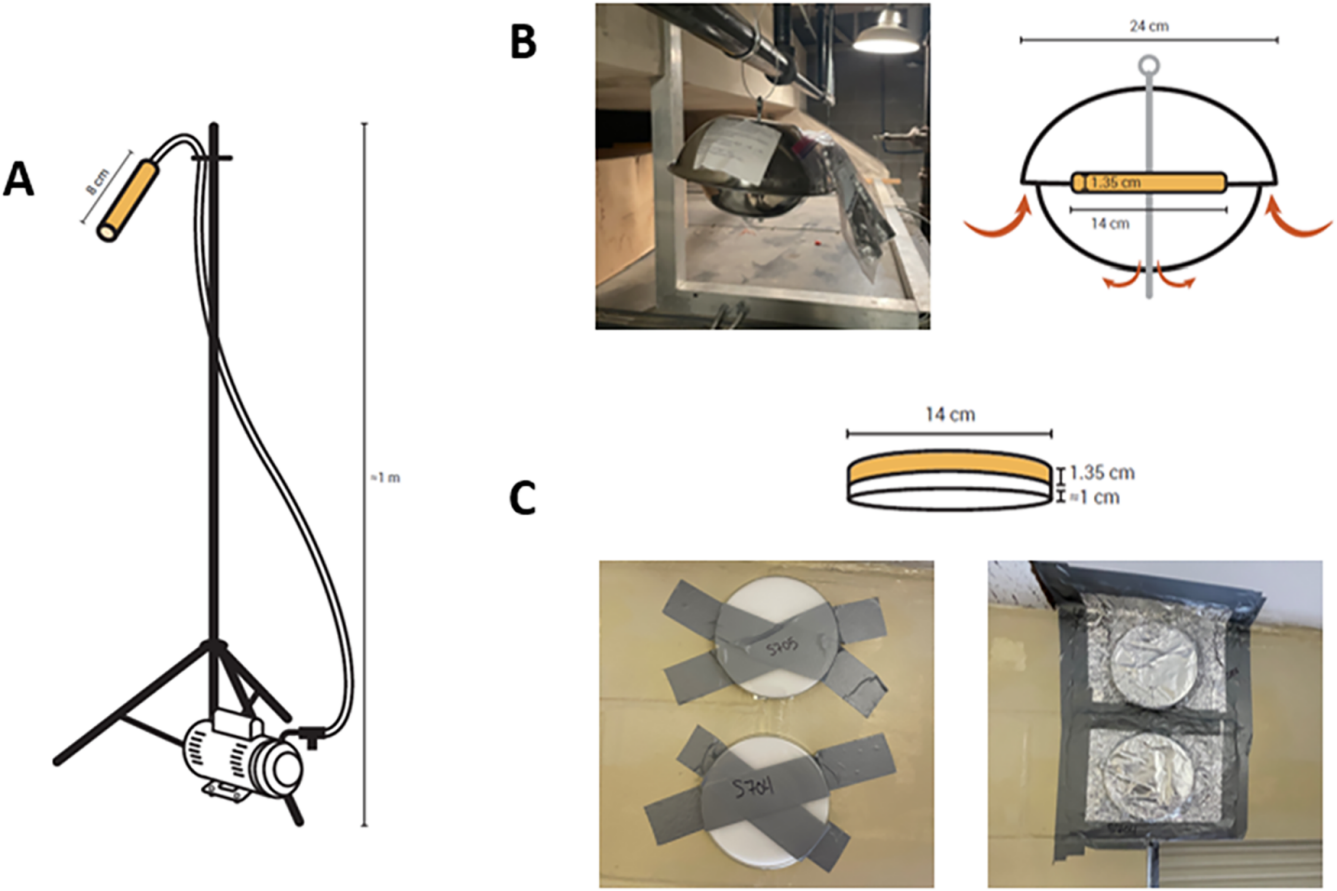
Airborne PCB data described in this paper originate from three sampler types: (A) low volume active air samplers analyzed for total PCBs (data reported by VT DEC), (B) polyurethane foam passive air samplers (PUF–PAS, this study) analyzed for all 209 PCB congeners, and (C) polyurethane foam passive emission samplers (PUF–PES, this study) also analyzed for all 209 PCB congeners.

In collaboration with Vermont state and school officials, we conducted a research study to measure PCB emissions from room surfaces in Vermont schools. Through this study, we identified building materials with the greatest contribution to PCBs in room air in Vermont schools and tested the hypothesis that airborne PCBs in school rooms are due to emissions from building materials containing Aroclors. We piloted field deployment of PUF–PES to measure direct emissions from building materials in an earlier study of one school^13^ and here we present the findings from a wide variety of surfaces in 16 schools across Vermont. This study reveals primary emission sources of PCBs in Vermont schools, which directly guides abatement and remediation by filling in the gap between measurements of PCBs in air and in solid materials. This study highlights the persistent risks associated with widespread use of hazardous chemicals in materials with a long lifespan.

## Methods

### Sampler Deployment

This study describes airborne PCBs collected using three types of samplers: low-volume active air samplers, polyurethane foam passive air samplers (PUF–PAS), and polyurethane foam passive emission samplers (PUF–PES). Low-volume active samplers were used by commercial laboratories contracted by the State of Vermont, Department of Environmental Conservation (VT DEC). Briefly, this method required a low-volume sampler to pull air through a sorbent cartridge containing polyurethane foam (PUF) at a rate of ∼5 L min^–1^ over a 24-h period. PCBs were then extracted from the PUF and analyzed by gas chromatography (GC) coupled with electron capture detector (ECD). Results were reported as total PCBs and Aroclor concentrations. The State of Vermont prioritized schools for testing based on factors including year of construction or renovation, age of youngest students, planned HVAC updates, planned construction, if prior PCB mitigation was completed, and free and reduced lunch percentages. Vermont’s consultants deployed samplers throughout school buildings, including offices and ancillary spaces. Whenever possible, the samplers were placed in the center of the room. Each school’s results are publicly available online via the VT DEC.^31^ The VT DEC organized these data, released them on their public Web sites, and provided the values reported as total PCB concentration (ng m^–3^). For this study, we evaluated PCB data reported by VT DEC as of July 18, 2024, which included 4800 air samples from 132 schools.^31^

PUF–PAS are widely used in research studies worldwide, notably for the Global Atmospheric Passive Sampling Network managed by Environment and Climate Change Canada.^34,35^ Our team previously used PUF–PAS for studies of PCBs in schools from Indiana, Iowa, and Vermont.^10,13,36,37^

PUF–PES were developed at the University of Iowa specifically to capture PCB emissions from building materials, including paint colorants, walls, floors, and cabinets.^8,13,38,39^ We have previously demonstrated the effectiveness of dual deployment of PUF–PAS and PUF–PES identifying emission sources in a single school.^13^ PUF–PES captures gross emissions, and this study does not specifically measure deposition.

PUF disks were cleaned prior to deployment using pressurized acetone and hexane, tightly wrapped in foil, placed in a Ziploc bag, and stored in a freezer at −10 **°**C until shipped. Sampling supplies were shipped overnight from the University of Iowa to Vermont and stored in boxes at room temperature until deployed. A unique identification code was given to each PUF after it was cleaned and recorded in a chain of custody as it was deployed. We deployed PUF–PAS and PUF–PES for 4-week periods throughout the schools. All surfaces were wiped with 75% alcohol wipes to remove dust and debris before PUF–PES were placed. Classrooms were our primary sampling site, but we also deployed samplers in gymnasiums, auditoriums, storage closets, boiler rooms, and utility rooms. At the end of the deployment period, samples were retrieved by DEC personnel, wrapped in clean foil, and shipped back to the University of Iowa where samples were stored in a −10 **°**C freezer until extracted. Additional details for extraction and instrument analysis methods are included in the Supporting Information along with equations for calculating PCB mass and effective volume.

### Material Selection and Sampling Strategy

Prior to sampler deployment in each school, we walked through the building with VT DEC personnel and school staff and visually inspected rooms for materials that potentially contained PCBs. VT DEC also provided material inventories and any previous air sampling results, if available, for each school. When data was available, we prioritized rooms with high concentrations and suspected PCB-containing materials. Along with the DEC, Vermont’s consultants and school staff also assisted our sampling strategy by pointing out suspect materials and specific locations in the building with potential sources. In addition to suspected sources, emissions from five common surfaces were sampled in all schools: carpet, concrete masonry unit (CMU) wall, cove base, drywall, and floor tile. Our PUF–PES is limited to flat surfaces; therefore we did not measure emissions from materials in corners or narrow fittings such as window caulking. We codeployed PUF–PAS in every room where we placed PUF–PES.

### Quality Assurance and Quality Control

We used several methods to assess the quality of the PUF–PAS and PUF–PES data. A limit of quantification (LOQ, ng per sample) was calculated for each congener and each school using PUF field blanks (*n* = 53) retained at the school during sample deployment. The LOQ was calculated as the upper limit of the 99% confidence interval of the log 10-transformed blank masses. Field blanks approximated a log-normal distribution. Congener masses are reported as measured and not replaced in instances where values are below LOQ. Accuracy of our methods was assessed through analysis of certified PCB concentrations in house dust standard reference material (SRM) from the National Institutes of Standards and Technology (NIST) sprinkled on PUF (NIST, SRM 2585, Gaithersburg, MD). Precision of our extraction method was assessed with surrogate standards and method blanks. The average total mass of method (*n* = 106) and field blanks were 8.9 and 9.3 ng, respectively, which was a negligible mass compared to what was measured in the PUF–PAS and PUF–PES. The average surrogate recovery ranged between 72–108%. We corrected sample masses for recoveries below 100%. The full data set of congener-specific measurements and quality control assessment is available at 10.25820/data.007328.^40^ Additional details of our method are included in the Supporting Information.

### Statistical Methods

We evaluated the differences in congener profiles using cosine theta (cos θ) and t-distributed stochastic neighbor embedding (t-SNE). Cos θ was used to compare each sample’s congener distribution to every other sample and to Aroclors. This approach quantitatively evaluates similarities in the complex congener signals of environmental and laboratory PCB measurements.^41,42^ We used t-SNE to visualize and interpret PCB congener signals. Like Principal Component Analysis (PCA) and cluster analyses–both of which have been used to examine PCB congener signals in environmental samples,^42–45^ t-SNE is a dimensionality reduction technique that maps high-dimensional data onto a low-dimensional space while maintaining the relationship of samples. We used t-SNE to identify groups of samples with similar profiles.^46^ t-SNE has been used across many fields including data science, machine learning, bioinformatics, and environmental science.^46,47^ To our knowledge, this study represents the first use of t-SNE to interpret PCB congener signals. Wilcoxon rank-sum test was used to assess the difference in median emissions between materials from schools with primary sources and schools without. We grouped emissions measurements from schools with primary sources and schools without, then calculated the median for each material. The significance threshold was *p* < 0.05. All analyses were performed using R version 4.3.1 (R Foundation, Vienna, Austria) and RStudio 2025.05.1 + 513 ″Mariposa Orchid″ Release (Posit PBC, Boston, Massachusetts).

## Results and Discussion

### Airborne PCBs in Vermont Schools

To assess occupant exposure to PCBs, the State of Vermont measured air concentrations using low-volume active samplers (Figure 1A). Results are reported as concentrations of total PCBs and of individual Aroclors, which ranged from nondetectable (ND) to 2600 ng m^–3^. Vermont’s concentration data indicated 38% of schools sampled so far have at least one exceedance of a SAL and 14% of schools sampled have at least one exceedance of an IAL. These results are consistent with nation-wide estimates of PCB use in caulking, which predict that out of approximately 48,000 schools constructed in the US between 1950 and 1980, 13,000–26,000 (27–54%) schools could have building materials that contain PCBs.^48^ Therefore, not every school will have exceedances, and measurements are required to confirm the presence of airborne PCBs in any individual school.^37^

To identify sources of PCB emissions from building materials and streamline corrective action, we deployed air and emission samplers in Vermont schools.^13,34,35^ Although the state obtained a large amount of air concentration data, air samplers do not elucidate specific sources that could inform remediation and abatement strategies. Therefore, in collaboration with the VT DEC, we collected 159 air samples (Figure 1B) and 182 emission samples (Figure 1C) from 16 schools (Table S4) and 98 school rooms.^40^

From over 4800 air measurements across 132 schools collected by this study and the State of Vermont, we reveal the extent, magnitude, and variability of PCBs in schools (Figure 2). Air concentrations measured by PUF–PAS ranged between 1.7 and 5700 ng m^–3^ (∑PCB). The arithmetic mean, geometric mean, and median of concentrations were 281, 48, and 59 ng m^–3^, respectively. Consistent with previous work in an Iowa school,^10^ we found substantial variation in PCB concentrations between rooms in the same school. Significant differences in concentrations within a building indicate a source may only be present in some parts of a school. Therefore, identifying the sources and understanding the reasons for the variation in air concentrations are crucial for making informed decisions about mitigation and remediation.

**Figure 2:**
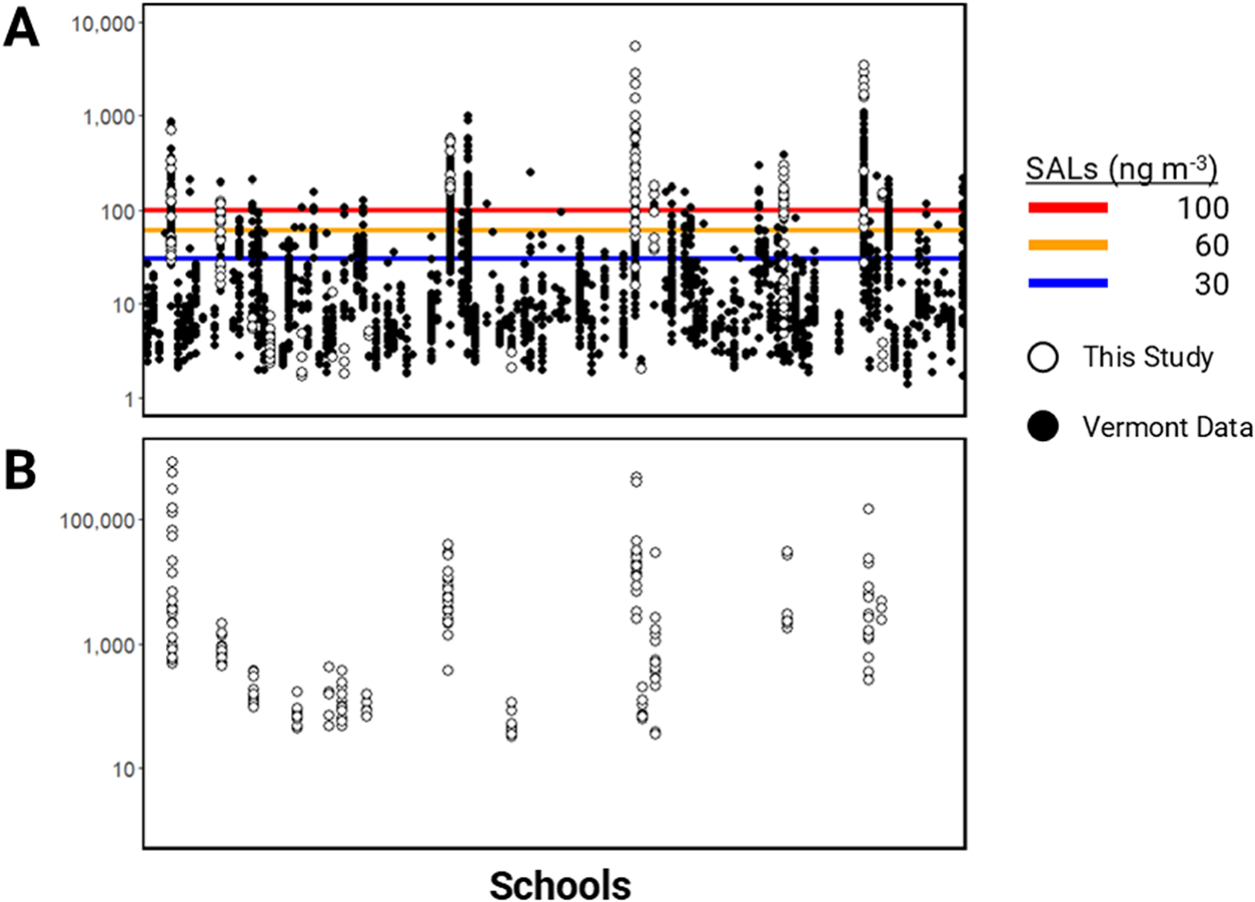
PCBs measured in Vermont schools (*n* = 132). (A) PCB air concentrations (ng m^–3^) as a sum of 209 PCBs (this study, open symbols) and reported by VT DEC as total PCBs only (black symbols). Vermont school action levels (SAL) are indicated by the colored lines: 30 ng m^–3^ for pre-kindergarten (blue), 60 ng m^–3^ for kindergarten–sixth grade (orange), and 100 ng m^–3^ for seventh grade–adult (red). (B) PCB emissions from school material surfaces measured as a sum of 209 PCBs (this study, ng m^–2^ d^–1^).

PCB concentrations in these schools are similar to concentrations reported in other schools and public buildings (Table 1).^10,14,15,36,37,49–52^ Concentrations in schools were higher than concentrations measured in North American and European homes and apartments (Table S15).^38,50,53–59^ Only two studies measured concentrations higher than our study in a public building (>6000 ng m^–3^)^52^ and schools (>10,000 ng m^–3^).^60^ Some of these studies calculated total PCB concentration using six (PCB 28, 52, 101, 138, 153, 180) or seven (PCB 28, 52, 101, 118, 138, 153, 180) indicator congeners multiplied by five. When we used this method to calculate concentration with our data, we overestimated concentrations by an average of 34%.

**Table 1:** Comparison of PCB Concentrations (ng m^–3^) from Vermont Schools and Previous Studies in Schools and Public Buildings

study	sampler	building type	min	median	mean	max	method
this study	PAS	school	1.7	59	281	5700	all congeners
this study	low-vol.	school	non-detect	15	52	2800	Aroclor analysis
Gabrio (Don) 2002 ^60^	low-vol.	school	181	NA	635	1587	six congeners times 5^a^
Gabrio (Wai) 2002	low-vol.	school	3060	NA	7490	10,655	six congeners times 5^a^
Gabrio (Neu) 2002	low-vol.	school	77	NA	3541	10,125	six congeners times 5^a^
Coghlan^49^	low-vol.	public building	<50	NA	NA	393	aroclor analysis
Kohler^52^	low-vol.	public building	<100	410	790	>6000	six congeners times 5^a^
Harrad^50^	PAS	public building	0.8	5.9	18.1	101.7	six congeners times 5^a^
Heinzow^51^	high-vol.	public building	715	NA	NA	2250	six congeners times 5^a^
MacIntosh^14^	low-vol.	school	299	432	533	1800	Aroclor analysis
Thomas^15^	low-vol.	school	<47.5	NA	NA	1005	Aroclor analysis
Ampleman^36^	PAS	school	0.4	NA	NA	160	all congeners
Marek^37^	PAS	school	0.5	17	41	194	all congeners
Bannavti^10^	PAS	school	1.24	12.9	15.2	39.2	all congeners

a ∑PCB = 5­(∑congeners 28, 52, 101, 138, 153, 180).

We found non-Aroclor PCBs in most samples, including PCB 11 (0.03–2.8 ng m^–3^), which are produced inadvertently during the manufacture of other products.^39,61–63^ PCB 11 was much higher inside schools than reported outdoor measurements around the US (max = 0.3 ng m^–3^)^64–66^ and similar to concentrations measured in Iowa residences. (0.1–2.17 ng m^–3^).^38^

The total concentration of PCB congeners (ΣPCB, ng m^–3^) we measured with PUF–PAS was statistically comparable to values reported by Vermont from the TO-10A method (Wilcoxon signed-rank test, *p* = 0.63) (Figure 3). It should be noted that the sample integration period for the passive samplers is much longer than the low volume active samplers, which could also explain differences in the results. We saw no evidence of bias between the low-vol. and PUF–PAS samples collected from the same rooms. Rooms that exceeded SALs exhibited much greater variability, likely due to sampler proximity to specific surface emission sources.^13^

**Figure 3:**
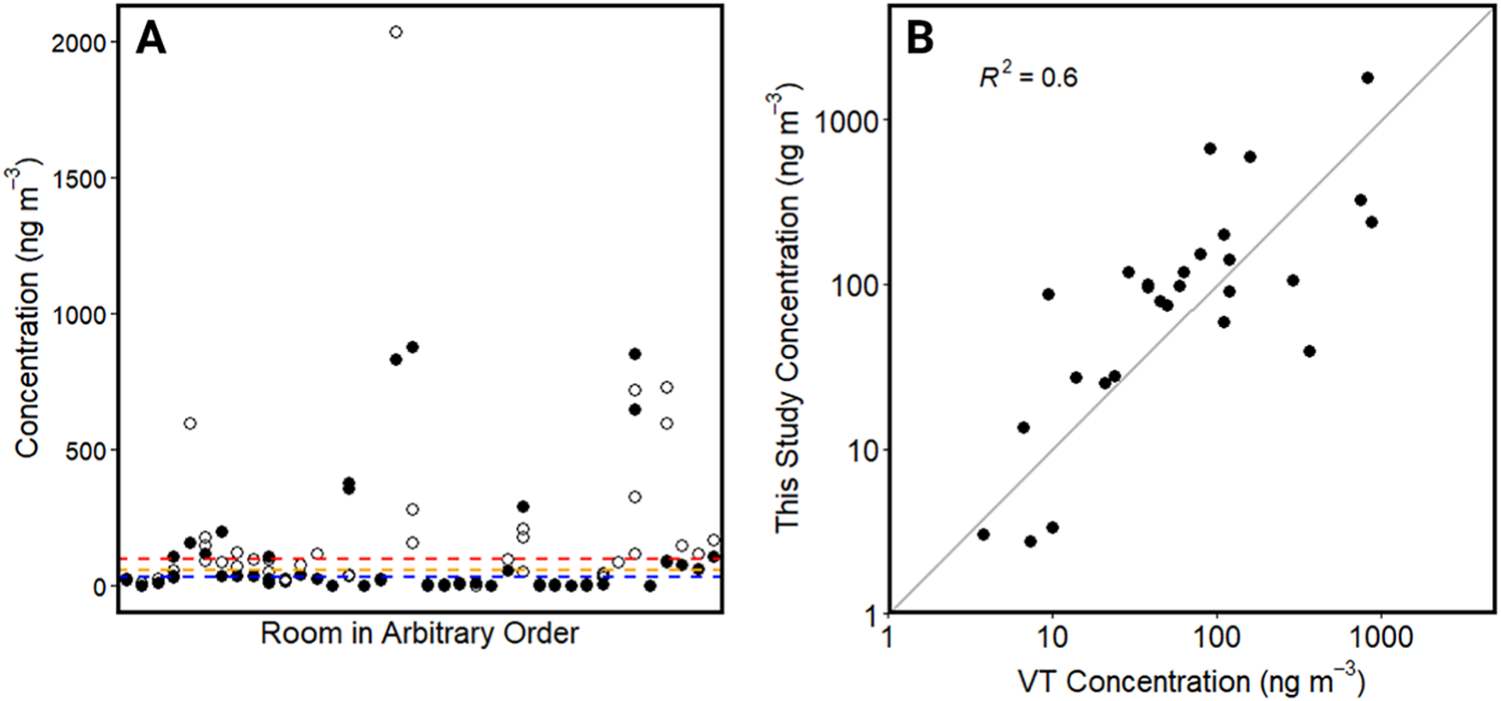
Thirty-two rooms across 14 schools were sampled by both VT DEC and this study. Airborne PCB concentrations were measured in the same school rooms but on different days. (A) Open circles represent findings from this study and filled circles are concentrations reported by VT DEC. Colored, dashed lines represent each SAL: 30 ng m^–3^ for pre-kindergarten (blue), 60 ng m^–3^ for kindergarten – sixth grade (orange), and 100 ng m^–3^ for seventh grade–Adult (red). (B) One-to-one plot comparing concentration measurements between this study and VT DEC.

### PCB Emissions from Building Materials

We measured PCB emissions from every surface we sampled. We placed 182 PUF–PES and measured direct PCB emissions from 23 types of surfaces in schools (Table S8). We found that emissions from three surface types collected from six different rooms were orders of magnitude greater than all other materials in the same classroom (Figure 4): fireproof coatings on steel columns (53,000–830,000 ng m^–2^ d^–1^), joint sealants on walls and ceilings (30,000–480,000 ng m^–2^ d^–1^), and a glass block window (30,000 ng m^–2^ d^–1^). Many studies have reported concentrations of PCBs in joint sealants and caulking.^11,15,49,67–69^ However, we are not aware of any studies that report concentrations of PCBs in fireproof coatings.

**Figure 4:**
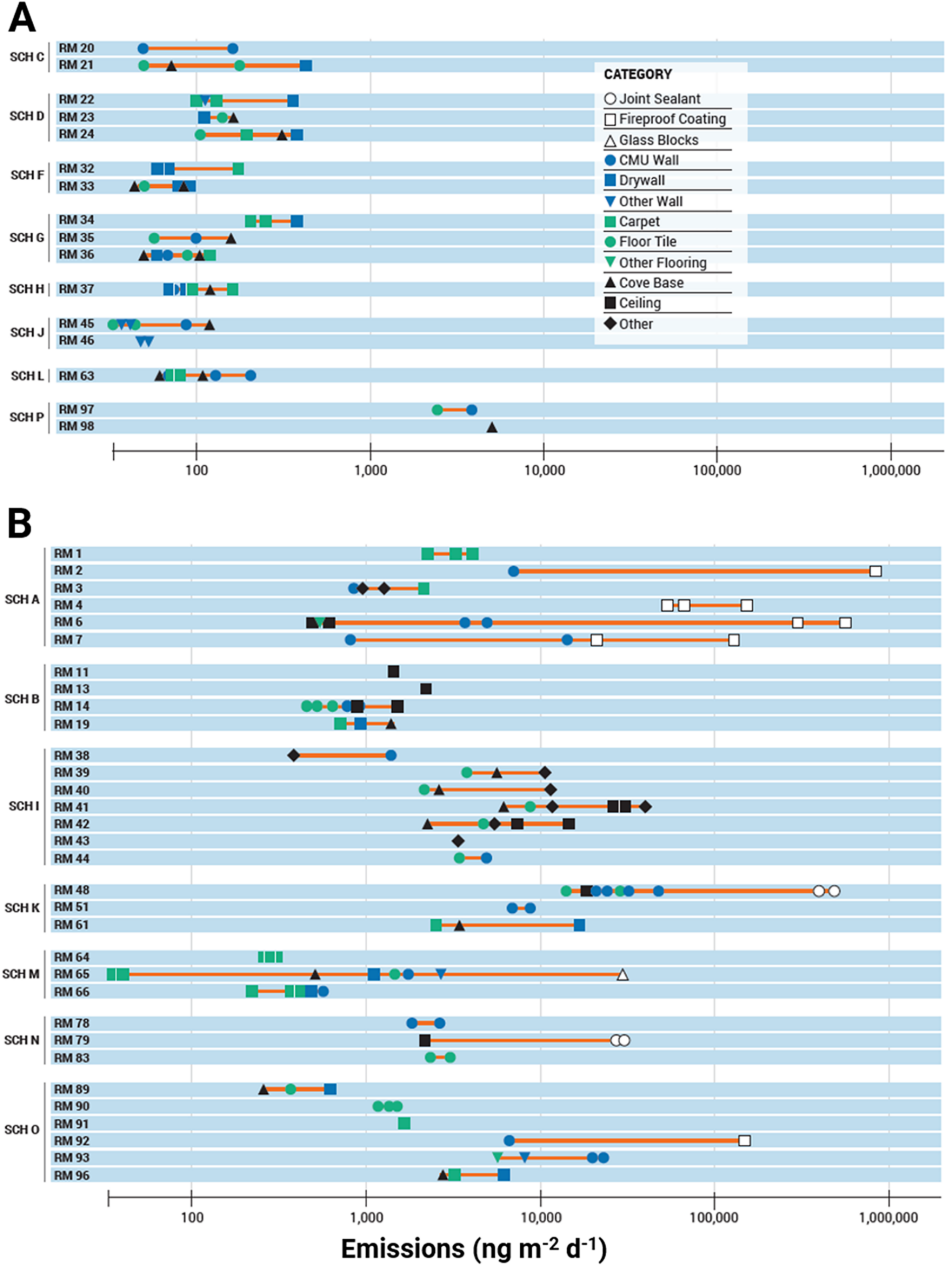
Symbols indicate the emission measured using the PUF–PES sampler and the category of the emission surface is described in the legend. Emissions are grouped by schools without a SAL exceedances based on VT DEC’s sampling (A) and schools with SAL exceedances (B). The school and room identifiers listed on the vertical axis are fully defined in the Supporting Information. The range of emissions measured in each room is linked with the red line represented on a log scale on the horizontal axis (ng m^–2^ d^–1^). Unfilled symbols indicate primary emission sources.

Few studies have reported emissions of PCBs from indoor surfaces. Herkert et al. reported ΣPCB emissions from surfaces in homes that ranged <LOQ to 33 ng m^–2^ d^–1^ off of finished cabinets.^38^ Bannavti et al. measured PCB emissions from surfaces in an office with known PCB contamination and reported a range of 1300 to 5000 ng m^–2^ d^–1^. Lyng et al. used a test cell to measure emissions from various surfaces in a school and reported a ΣPCB_6_ emission range of 3.17 to 683.8 ng m^–2^ d^–1^ prior to remediation.^70^ Emissions we measured in Vermont schools were much higher than what has been reported previously.

The other 20 surface types we assessed included CMU wall, vinyl floor tiles, drywall, carpet, cove base, and brick walls (33–45,000 ng m^–2^ d^–1^). We are not aware of any reports that Aroclors were added to these materials, yet emissions measured from some surfaces are as high as primary sources (Table S9).We hypothesize that these surfaces are affected by proximity to high concentrations in solid materials or high emissions from primary sources. Emissions that result from diffusion in the solid material surrounding a Aroclor source (like caulking) followed by emission are called secondary sources. Emissions due to many years of atmospheric deposition are called tertiary emissions.

PCBs volatilize and redeposit repeatedly, and this well-known phenomenon explains their global distribution to remote places where Aroclors and non-Aroclors were never produced or used.^71–78^ This same phenomenon is true indoors and causes the tertiary emissions we measured from walls, flooring and ceilings. Although there are few studies that focus on PCBs, this repartitioning effect has been demonstrated for other SVOCs.^59,79,80^ Previous studies have examined transport of PCBs from primary sources onto building materials and dust but do not examine reemission and redeposition.^59,81^ In rooms where we found PCB emissions from fireproof coating, joint sealants, and/or glass block windows, we also found elevated emissions from CMU wall, vinyl floor tiles, drywall, carpet, cove base, brick walls and other room surfaces (Figure 4). We measured elevated emissions throughout SCH I but did not find a clear primary source like glass block windows or fireproofing. This school had window and door frame caulking that was suspected to be a source, but we were unable to measure emissions from these materials with our PUF–PES. We then assessed the range of emissions in rooms that did not contain fireproof coating, expansion joint sealants, and/or glass block windows and found emissions from flooring and walls were much lower in schools that did not have an identified primary source. (Figure 4) When we compared emissions from similar materials between schools that had SAL exceedances and schools without exceedances, the range of emissions was much greater in schools with airborne PCB concentrations above the SAL (SCH A, SCH B, SCH I, SCH K, SCH M SCH N, SCH O). Therefore, when a primary source is present, it causes higher emissions from all other surfaces in the room, due to continuous volatilization and deposition of PCBs within the school rooms over many decades (Figure 5).

**Figure 5:**
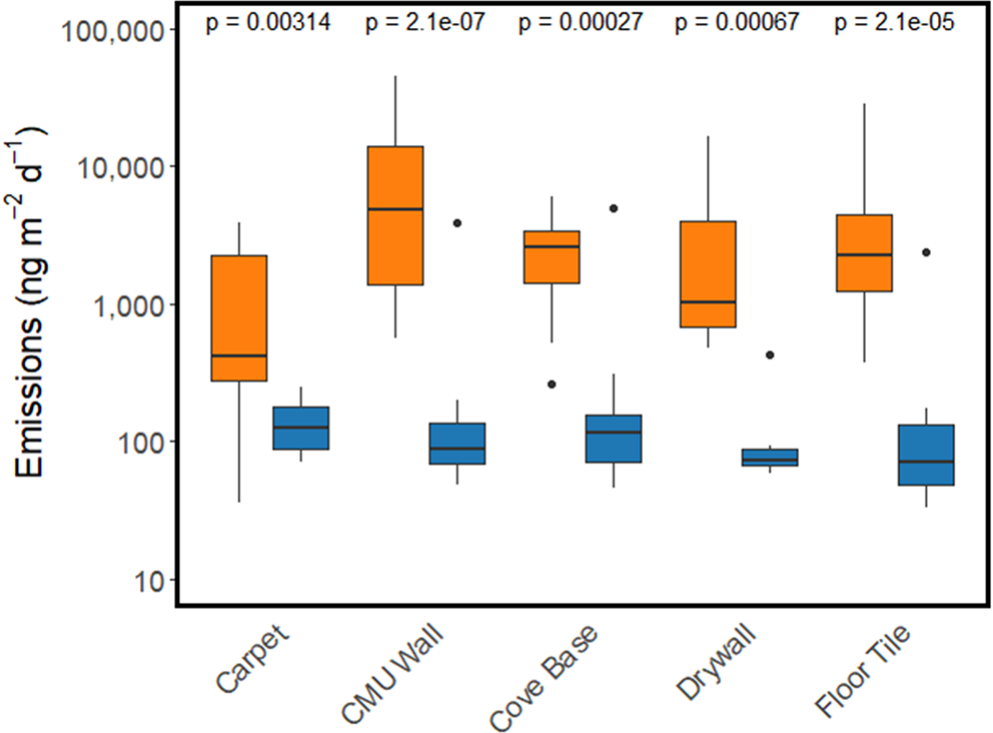
We found a significant difference (Wilcoxon rank-sum test) between emissions from each surface measured in schools with a primary source present (orange boxes) and schools without an identified primary source (blue boxes). *P*-values for each surface are shown above the respective boxes.

### Congener Analyses

To inform risk assessments and identify the most prevalent mixtures of PCBs in schools, we measured all 209 PCBs in our samples and evaluated the similarity of the congener distributions in each sample to that in Aroclors. We confirmed that the commercial Aroclor mixtures, mainly Aroclor 1254, installed when the room was constructed or remodeled are still present in those materials and are the primary sources of airborne PCBs. Aroclors were the only commercial mixtures of PCBs that were sold in the US and were produced by Monsanto Company, which distributed about ten distinct mixtures of PCB congeners throughout the country.^82^ We identified Aroclors by comparing the PCB congener distributions in our measurements with published congener distributions for Aroclors.^83^ We also repeated the same comparison after normalizing each congener to its saturation vapor pressure to account for the differences in relative volatility of the PCB congeners (Figure S3).^13,83^ For this analysis, we used cosine similarity (cos θ) to describe the similarity of sample profiles with a number between 0 (completely dissimilar) and 1 (identical). Although the effect is small, the similarity between samples and Aroclors generally increased after normalizing for vapor pressure for these Aroclors. Overall, we found no evidence of environmental weathering of PCB congeners that could cause us to mis-identify the Aroclor used in schools.^13^ Using this approach, we found evidence of many Aroclors in the emissions and air samples from these schools including Aroclors 1016/1242, 1232, 1248, 1254, 1260, and 1262 (cos θ > 0.9).

We also detected PCB emissions that originated from non-Aroclors. Paint, colored construction paper, inkjet printers, finger paints, and colored chalk may contain PCBs produced as byproducts of contemporary chemical manufacturing processes, and these PCBs may be released into rooms and subsequently deposit and reemit from the surfaces we studied.^62,63,84,85^ Although these Aroclor- and non-Aroclor-containing materials in schools may pose a risk of airborne exposure to occupants, our findings show that high air concentrations and emissions in schools were due to Aroclor sources.

Overall, samples within the same schools are most similar to each other (cos θ > 0.9). The most common match was to either the original Aroclor 1254 or its vapor-pressure adjusted congener distribution. Air samples tended to be most similar to the vapor-pressure adjusted distribution. In contrast, the highest emission sources we measured, such as the fireproof coating, were most similar to the original Aroclor 1254 profile. We also found a distinct difference between samples from schools with major sources and schools that do not exceed SALs. Congener profiles of air and emission samples collected in schools that exceeded SALs are most similar to one or more Aroclors.

To visualize patterns in congener distributions between schools and assess differences in profiles from school to school, we used t-SNE, a dimensionality reduction technique that maps high-dimensional data onto a low-dimensional space while maintaining the relationships among samples.^46,47^ The t-SNE analysis was consistent with the cosine similarity findings: emissions and air samples exhibited great similarity to Aroclors. Moreover, samples deployed in the same school tend to have similar profiles and therefore cluster together. The 3D t-SNE yields five clusters (Figure 6): (1) samples similar to Aroclor 1254 and vapor-pressure adjusted Aroclor 1260; (2) samples similar to the vapor-pressure adjusted 1254 profile; (3) samples similar to Aroclor 1232, 1248, 1260, and 1262; (4) samples similar to Aroclor 1016/1242; and (5) samples that do not resemble any Aroclors (Table S11). Samples in cluster 4 (SCH B) strongly resemble Aroclor 1016/1242, a signal from light ballasts. However, we cannot confirm if this signal was due to present light ballasts or residuals from previously removed light ballasts. Samples in cluster 5 had detectable levels of PCBs at much lower concentrations and emissions compared to other samples. Therefore, Aroclors are the main, and most concerning, source of airborne PCBs in schools despite a ban on their production in the late 1970s.

**Figure 6:**
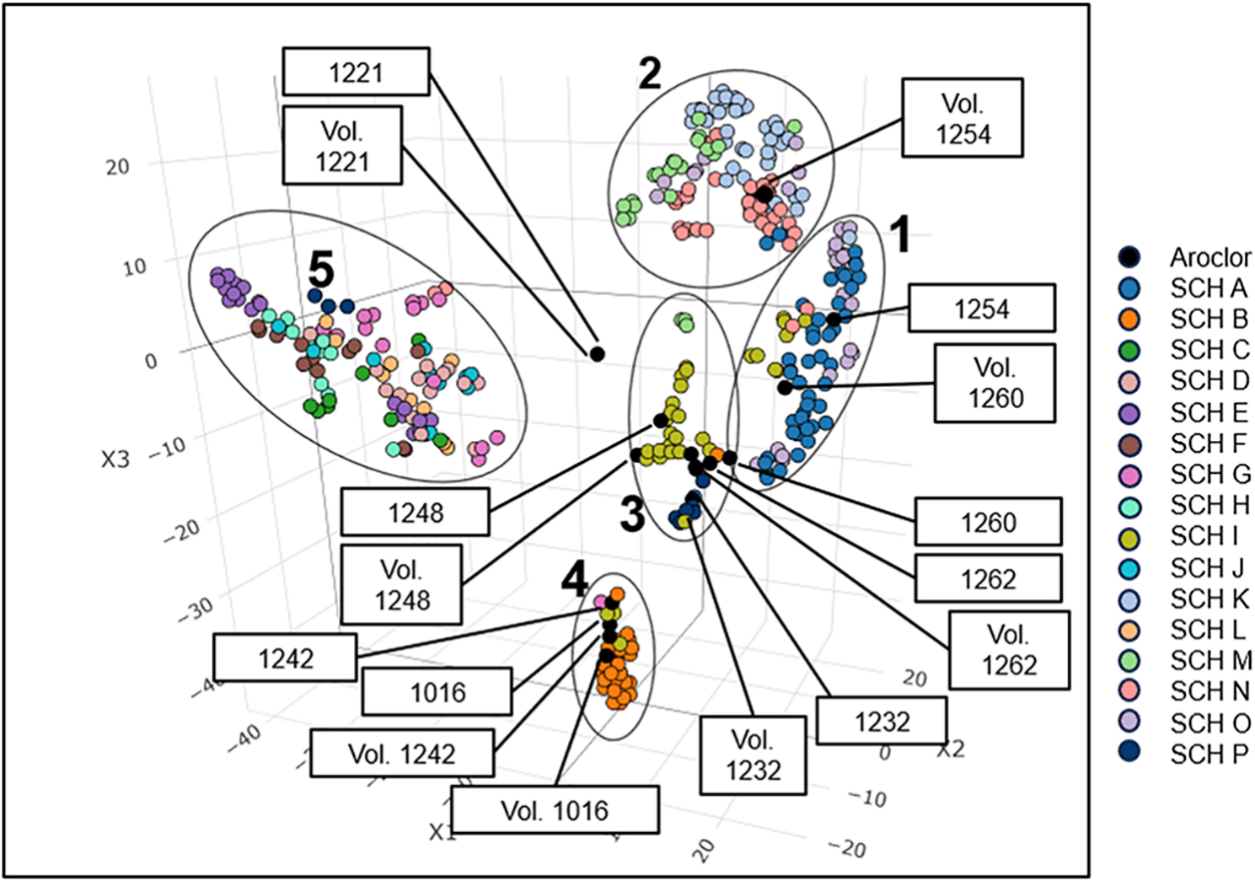
Three-D t-SNE visualizing the similarities between sample congener distributions, both air and emissions, and Aroclor congener distributions. Each dot represents a sample and each color represents a different school. Black dots represent Aroclors. Samples collected within the same school tend to be most similar to each other. Aroclors are present throughout most clusters either as the Aroclor or vapor-pressure adjusted Aroclor (Vol.) distribution. Clusters are numbered 1–5 and discussed in the text.

Itemization of approved or intended uses of Aroclors in the United States are remarkably vague, imprecise, or unconfirmed. For example, the ATSDR Toxicological Profile of PCBs presented a summary of Aroclors and their specific uses.^86^ Flame retardant spray was not tabulated as a potential use of Aroclor 1254 or any other Aroclor. Window glazing and building architectural joint sealants were also not specifically mentioned, although ‘sealant and caulking compounds’ were listed only with the use of Aroclor 1254.^86^ Erickson and Kaley also summarized Monsanto’s uses of Aroclors in a variety of products, many of which include PCBs as an additive that was mixed into the existing product or material.^82^ The marketing and sale of Aroclors as additives complicate the identification and tracking of PCB-containing materials because these uses are not documented. In their discussion of flame retardant materials, Erickson and Kaley focused on electrical equipment, rigid polyurethane foams, and fiberboards and did not specifically mention the spray insulation we found in schools. The total sales, frequency of use, and preparation of Aroclor-containing spray insulation is unknown. Aroclors may have been added to construction materials just prior to application in the school or may have been added by the manufacturer and vendor of the product. Clearly, an inventory of Aroclors in building materials, constructed from direct measurement rather than industry reports, is needed. To reduce children and staff exposures in schools, these materials must be removed from the building or encapsulated, or they will continue to emit harmful PCBs.

### PCB Exposure

We found PCB concentrations in schools at levels that pose an elevated risk of developing adverse health effects. To characterize the potential public health risk of exposure to PCBs in schools, we quantified all 209 PCBs in every sample. This comprehensive approach allowed us to assess cancer risk separately for dioxin-like and nondioxin-like PCBs. Congeners that target molecular pathways associated with endocrine signaling, neuronal signaling, astrocyte metabolism, adipocyte function, and carcinogenesis were measured at high concentrations throughout schools with a major source of PCBs.^87–95^ In some rooms, the calculated exposure from the sum of PCB congeners exceeds the US EPA noncancer reference dose for Aroclor 1254, which presents concern for immunological and other noncancer outcomes in students and staff (eqs S9 and S10).^96–98^ We frequently detected dioxin-like PCBs 105, 118, and 156 + 157 in school air,^40^ which are among the 12 dioxin-like congeners and are designated as carcinogens by the World Health Organization (WHO).^95,99,100^ PCB mixtures, including nondioxin-like congeners, are also carcinogens.^101^ Consequently, school staff working in these buildings for 30 years have an estimated excess lifetime cancer risk from inhalation of both dioxin-like and nondioxin-like PCBs that ranges from 1.3 × 10^–8^ to 1.7 × 10^–4^ for central tendency exposure, and 2.8 × 10^–8^ to 3.8 × 10^–4^ for reasonable maximum exposure (Table S12). To establish acceptable exposure levels, the State of Vermont sets the target incremental increase in lifetime cancer risk to 1 excess cancer per million people exposed (1 × 10^–6^).^102^ The US EPA sets the target risk between 1 × 10^–6^ and 1 × 10^–4^.^103^ Therefore, some people at Vermont schools may be exposed to PCBs at levels that exceed the target cancer risk, as defined by the State of Vermont and the US EPA. The congener analysis shows that in the schools we sampled, nondioxin-like congeners account for a significant portion of the cancer risk.

## Implications of Direct Emissions Sampling in Schools

To date, this study is the largest assessment of PCB emissions from different surfaces in schools. Using this application of our emission samplers, we identified significant emissions of toxic Aroclor mixtures in schools, particularly from three materials: joint sealants, glass block windows, and fireproof coatings (all of which were large emitters of Aroclor 1254). We also showed that air measurements collected using a low-volume active sampler and total Aroclor PCB analysis is comparable to our research methods. Therefore, the low-vol. Aroclor analysis approach is an effective and practical option for assessing PCB air concentrations in public buildings.

Our ability to measure emissions directly from primary sources has implications for remediation decision making in schools. We have informed schools of which materials contributed the most to airborne PCBs and expedited corrective action. We also showed how much variability exists not only from school to school, but even from room to room. Therefore, codeployment of PUF–PAS and PUF–PES accurately and effectively provides useful results for remediation decision making.

As a practical matter, this study identifies priorities for action. Because this study showed that Aroclors are the major source of airborne PCBs in these schools, we recommend consideration of all schools built before 1980 and inspection of rooms for materials known to contain Aroclors. This recommendation includes previously reported sources not measured in this study such as light ballasts. We found Aroclor-emitting caulks and sealants were most often located in expansion joints or between concrete panels and walls in this study. Aroclor-emitting fireproofing appeared as textured coatings on steel columns and other structural elements. The building’s age and the presence of these materials can indicate potential for elevated air concentrations and exposure risks. Removal of primary sources are a priority for reducing occupant exposure. This study identified three primary PCB sources in schools. However, more work must still be done to fully characterize all potential emission sources in schools.

## Supplementary Material



## Data Availability

The full data set is available (DOI https://pubs.acs.org/doi/10.25820/data.007328).

## Notes

Additional method details including extraction conditions; instrument settings; chemical structure identification; quality control data including analysis of standard reference material, surrogate recoveries, and congener-specific limits of quantification; effective sampling volume calculations; emission model parameters; Aroclor 1254 profiles; and cosine similarity analyses (PDF)
