# Supplementary material for: Widespread Emissions of Polychlorinated Biphenyls from Building Materials in Vermont Schools
Source: Environ Sci Technol. 2026 Feb 3;60(6):4966–77. doi: 10.1021/acs.est.5c10939 (PMC12918525; doi:10.1021/acs.est.5c10939)
Supplement: Supplementary file 1 [file es5c10939_si_001.pdf]

Supporting Information

Widespread emissions of polychlorinated biphenyls from building materials in Vermont schools

Authors. Jason B.X. Hua[1], Rachel F. Marek[1], Michael P. Jones[2], Trevor D. Erb[1], Sarah C. Owen[3], and Keri C. Hornbuckle[1]*

[1]Department of Civil and Environmental Engineering, IIHR-Hydroscience and Engineering, The University of Iowa, Iowa City, IA, 52242, USA

[2]Department of Biostatistics, The University of Iowa, Iowa City, IA, 52242, USA

[3]Division of Environmental Health, Vermont Department of Health; Waterbury, 05671, USA.

*Corresponding author keri-hornbuckle@uiowa.edu Ph (319-384-0789)

Data generated in this research are available at https://doi.org/10.25820/data.007328.[1]

Number of pages: 30

Number of tables: 15

Number of figures: 5

Method and Materials

PUF Extraction

We used accelerated solvent extraction (Thermo Fisher Scientific Dionex ASE 350), with acetone and hexane (1:1 v/v) to extract PCBs from PUF samples (Tisch Environmental, Cleves, OH, Part # TE-1014). During ASE preparation, 9.84 ng of surrogate standard 13C labeled PCBs: 3, 15, 31, 52, 118, 153, 180, 194, 206, and 209, (Wellington Laboratories) were added to the PUF to allow for corrections due to analytical losses and variability. Recoveries of 13C labeled surrogate standards were used to correct masses of corresponding homologs (**Table S1**). Turbulent evaporation with nitrogen (Biotage TurboVap II Automated Solvent Evaporation System) was used to concentrate extracts. Extracts were cleaned by sulfuric acid silica gel columns. Samples were then concentrated again, transferred to 2 mL glass autosampler vials, and spiked with 10.06 ng of internal standard d-PCB 30 (2,4,6-trichlorobiphenyl-2',3',4',5',6'-d5, C/D/N Isotopes) and internal standard PCB 204 (2,2',3,4,4',5,6,6'-octachlorobiphenyl, AccuStandard).

Table S1. Ten 13C labeled PCBs were used as surrogate standard. Each standard was used to correct masses of the corresponding homolog.

Surrogate Standard	Homolog
13 C 3	Monochlorinated
13 C 15	Dichlorinated
13 C 31	Trichlorinated
13 C 52	Tetrachlorinated
13 C 118	Pentachlorinated
13 C 153	Hexachlorinated
13 C 180	Heptachlorinated
13 C 194	Octachlorinated
13 C 206	Nonachlorinated
13 C 209	Decachlorinated

Instrument Parameters

The GC (Agilent 7000B Triple Quad with Agilent 7890A GC, Agilent 7693 autosampler, and multi-mode inlet) was equipped with a Supelco SPB-Octyl capillary column (Poly (50% n-octyl/50% methyl siloxane, 30 m × 0.25 mm ID, 0.25 µm film thicknesses)) with UHP helium as the carrier gas (constant flow 0.8 mL/min) and quench gas (2.25 mL/min) and UHP nitrogen as the collision gas (1.5 mL/min). The GC inlet operated at the following conditions: initial temperature 45 °C, initial time 0.06 min, ramp 600 °C min-1 to inlet temperature 325 °C. The GC oven temperature program was 45 °C for 2 min, 45 to 75 °C at 100 °C min-1 and hold for 5 min, 75 to 150 °C at 15 °C min-1 and hold for 1 min, 150 to 280 at 2.5 °C min-1 and final hold 6 min (total run time 71.3 min). Congener identity according to elution was assigned following the USEPA Method 1668C (**Table S2**).[2] The MS transfer line temperature was held at 280 °C. The triple quadrupole MS electron ionization source was set to 250 °C, the dwell time 60 ms, and collision energy 25 V.

Table S2. Congener numbers and corresponding structures of all 209 PCBs in this study according to US EPA Method 1668C.

PCB#	Structure	PCB#	Structure	PCB#	Structure
1	2	44	2,2’,3,5’	87	2,2’,3,4,5’
2	3	45	2,2’,3,6	88	2,2’,3,4,6
3	4	46	2,2’,3,6’	89	2,2’,3,4,6’
4	2,2’	47	2,2’,4,4’	90	2,2’,3,4’,5
5	2,3	48	2,2’,4,5	91	2,2’,3,4’,6
6	2,3’	49	2,2’,4,5’	92	2,2’,3,5,5’
7	2,4	50	2,2’,4,6	93	2,2’,3,5,6
8	2,4’	51	2,2’,4,6’	94	2,2’,3,5,6’
9	2,5	52	2,2’,5,5’	95	2,2’,3,5’,6
10	2,6	53	2,2’,5,6’	96	2,2’,3,6,6’
11	3,3’	54	2,2’,6,6’	97	2,2’,3’,4,5
12	3,4	55	2,3,3’,4’	98	2,2’,3’,4,6
13	3,4’	56	2,3,3’,4’	99	2,2’,4,4’,5
14	3,5	57	2,3,3’,5	100	2,2’,4,4’,6
15	4,4’	58	2,3,3’,5’	101	2,2’,4,5,5’
16	2,2’,3	59	2,3,3’,6	102	2,2’,4,5,6’
17	2,2’,4	60	2,3,4,4’	103	2,2’,4,5’,6
18	2,2’,5	61	2,3,4,5	104	2,2’,4,6,6’
19	2,2’,6	62	2,3,4,6	105	2,3,3’,4,4’
20	2,3,3’	63	2,3,4’,5	106	2,3,3’,4,5
21	2,3,4	64	2,3,4’,6	107	2,3,3’,4’,5
22	2,3,4’	65	2,3,5,6	108	2,3,3’,4,5’
23	2,3,5	66	2,3’,4,4’	109	2,3,3’,4,6
24	2,3,6	67	2,3’,4,5	110	2,3,3’,4’,6
25	2,3’,4	68	2,3’,4,5’	111	2,3,3’,5,5’
26	2,3’,5	69	2,3’,4,6	112	2,3,3’,5,6
27	2,3’,6	70	2,3’,4’,5	113	2,3,3’,5’,6
28	2,4,4’	71	2,3’,4’,6	114	2,3,4,4’,5
29	2,4,5	72	2,3’,5,5’	115	2,3,4,4’,6
30	2,4,6	73	2,3’,5’,6	116	2,3,4,5,6
31	2,4’,5	74	2,4,4’,5	117	2,3,4’,5,6
32	2,4’,6	75	2,4,4’,6	118	2,3’,4,4’,5
33	2’,3,4	76	2’,3,4,5	119	2,3’,4,4’,6
34	2’,3,5	77	3,3’,4,4’	120	2,3’,4,5,5’
35	3,3’,4	78	3,3’,4,5	121	2,3’4,5’,6
36	3,3’,5	79	3,3’,4,5’	122	2’,3,3’,4,5
37	3,4,4’	80	3,3’,5,5’	123	2’,3,4,4’,5
38	3,4,5	81	3,4,4’,5	124	2’,3,4,5,5’
39	3,4’,5	82	2,2’,3,3’,4	125	2’,3,4,5,6’
40	2,2’,3,3’	83	2,2’,3,3’,5	126	3,3’,4,4’,5
41	2,2’,3,4	84	2,2’,3,3’,6	127	3,3’,4,5,5’
42	2,2’,3,4’	85	2,2’,3,4,4’	128	2,2’,3,3’,4,4’
43	2,2’,3,5	86	2,2’,3,4,5	129	2,2’,3,3’,4,5

Table S2 continued

PCB#	Structure	PCB#	Structure	
130	2,2’,3,3’,4,5’	171	2,2’,3,3’,4,4’,6	
131	2,2’,3,3’,4,6	172	2,2’,3,3’,4,5,5’	
132	2,2’,3,3’,4,6’	173	2,2’,3,3’,4,5,6	
133	2,2’,3,3’,5,5’	174	2,2’,3,3’,4,5,6’	
134	2,2’,3,3’,5,6	175	2,2’,3,3’,4,5’,6	
135	2,2’,3,3’,5,6’	176	2,2’,3,3’,4,6,6’	
136	2,2’,3,3’,6,6’	177	2,2’,3,3’,4’,5,6	
137	2,2’,3,4,4’,5	178	2,2’,3,3’,5,5’,6	
138	2,2’,3,4,4’,5’	179	2,2’,3,3’,5,6,6’	
139	2,2’,3,4,4’,6	180	2,2’,3,4,4’,5,5’	
140	2,2’,3,4,4’,6’	181	2,2’,3,4,4’,5,6	
141	2,2’,3,4,5,5’	182	2,2’,3,4,4’,5,6’	
142	2,2’,3,4,5,6	183	2,2’,3,4,4’,5’,6	
143	2,2’,3,4,5,6’	184	2,2’,3,4,4’,6,6’	
144	2,2’,3,4,5’,6	185	2,2’,3,4,5,5’,6	
145	2,2’,3,4,6,6’	186	2,2’,3,4,5,6,6’	
146	2,2’,3,4’,5,5’	187	2,2’,3,4’,5,5’,6	
147	2,2’,3,4’,5,6	188	2,2’,3,4’,5,6,6’	
148	2,2’,3,4’,5,6’	189	2,3,3’,4,4’,5,5’	
149	2,2’,3,4’,5’,6	190	2,3,3’,4,4’,5,6	
150	2,2’,3,4’,6,6’	191	2,3,3’,4,4’,5’,6	
151	2,2’,3,5,5’,6	192	2,3,3’,4,5,5’,6	
152	2,2’,3,5,6,6’	193	2,3,3’,4’,5,5’,6	
153	2,2’,4,4’,5,5’	194	2,2’,3,3’,4,4’,5,5’	
154	2,2’,4,4’,5’,6	195	2,2’,3,3’,4,4’,5,6	
155	2,2’,4,4’,6,6’	196	2,2’,3,3’,4,4’,5,6’	
156	2,3,3’,4,4’,5	197	2,2’,3,3’,4,4’,6,6’	
157	2,3,3’,4,4’,5’	198	2,2’,3,3’,4,5,5’,6	
158	2,3,3’,4,4’,6	199	2,2’,3,3’,4,5,5’,6’	
159	2,3,3’,4,5,5’	200	2,2’,3,3’,4,5,6,6’	
160	2,3,3’,4,5,6	201	2,2’,3,3’,4,5’,6,6’	
161	2,3,3’,4,5’,6	202	2,2’,3,3’,5,5’,6,6’	
162	2,3,3’,4’,5,5’	203	2,2’,3,4,4’,5,5’,6	
163	2,3,3’,4’,5,6	204	2,2’,3,4,4’,5,6,6’	
164	2,3,3’,4’,5’,6	205	2,3,3’,4,4’,5,5’,6	
165	2,3,3’,5,5’,6	206	2,2’,3,3’,4,4’,5,5’,6	
166	2,3,4,4’,5,6	207	2,2’,3,3’,4,4’,5,6,6’	
167	2,3’,4,4’,5,5’	208	2,2’,3,3’,4,5,5’,6,6’	
168	2,3’,4,4’,5’,6	209	2,2’,3,3’,4,4’,5,5’,6,6’	
169	3,3’,4,4’,5,5’			
170	2,2’,3,3’,4,4’,5			

Table S3. PCB precursor and product masses of unlabeled and deuterated calibration standards used in multiple reaction monitoring (MRM) mode on the triple quadrupole mass spectrometer[α].

Cl homolog	Precursor Mass	Product Mass
mono	188	153.1
di	222	152.1
tri	256	186
tetra	291.9	222
penta	325.9	255.9
hexa	359.8	289.9
hepta	393.8	323.9
octa	429.8	359.8
nona	463.7	393.8
deca	497.7	427.7
D5 tri	261	191.1
13C mono	200.1	165.1
13C di	234	164.1
13C tri	268	198.1
13C tetra	304	234
13C penta	337.9	268
13C hexa	371.9	301.9
13C hepta	405.8	335.9
13C octa	441.8	371.9
13C nona	475.8	405.8
13C deca	509.7	439.8

[α]Unlabeled standards were from AccuStandard, New Haven, CT, USA. Deuterated PCB 30 was from C/D/N Isotopes, Pointe-Claire, QC, Canada. 13C labeled standards were from Wellington Laboratories, Guelph, Ontario, Canada.

Congener Mass Calculation

We measured concentrations and emissions of airborne PCBs for each of the 209 PCBs as 173 congener or coeluting congener groups and the sum of PCBs (∑PCBs). Peak areas were transformed into mass using a congener-specific relative response factor (RRF). The RRF for each congener, *i*, was calculated as:

$$RRF_i = \frac{m_{i,cal} \,/\, A_{i,cal}}{m_{is,cal} \,/\, A_{is,cal}} \qquad (eq\ S1)$$

where

$m_{i,cal}$ = *the known mass of target congener in the calibration standard*

$A_{i,cal}$ = *the measured peak area of the target congener in the calibration standard*

$m_{is,cal}$ = *the known mass of the internal standard in the calibration standard*

$A_{is,cal}$ = *the measured peak area of the internal standard in the calibration standard*

Congener masses were then calculated as follows:

$$m_{i,S} = RRF_i * A_{i,S} * \frac{m_{is,S}}{A_{is,S}} \quad (eq\ S2)$$

where
$m_{i,S}$ = the calculated mass of target congener in the sample
$A_{i,S}$ = the measured peak area of the target congener in the sample
$m_{is,S}$ = the known mass of the internal standard in the sample
$A_{is,S}$ = the measured peak area of the internal standard in the sample

Determining Sample Effective Volume

Different sampling rates (~1 m^3 d^{-1}) and effective volume calculations (usually ~30 m^3) were made for each school and varied depending on the deployment time (~ 30 days) and room temperature. In some schools, we measured temperatures hourly with a data logger (Testo 174T) and averaged our measurements for this calculation.[3]

The variable K_{PUF} is calculated by the empirical equation (Shoeib[4]):

$$\log K_{\mathrm{PUF}} = 0.6366 \log K_{OA} - 3.1774 \quad (eq\ S3)$$

where K_{OA} is calculated by (Herkert[5]):

$$\log K_{\mathrm{OA(T)}} = \log K_{OA(25°\mathrm{C})} - \frac{\Delta U_{OA}}{2.303 * R}\left(\frac{1}{T} - \frac{1}{298.15}\right) \quad (eq\ S4)$$

where T is the temperature (K), ΔU_{OA} is the internal energy of octanol-air transfer (J mol^{-1}), R is the gas constant (J mol^{-1} K^{-1}).

The sampling rate, R_s, is congener specific and calculated by:

$$R_{\mathrm{s}} = \left(f_{\mathrm{on}}\sqrt{WS_{\mathrm{on}}} + f_{\mathrm{off}}\sqrt{WS_{\mathrm{off}}}\right)\left(\frac{1}{\sqrt[3]{\mathrm{MW}}}\right)10^{(0.0012T+c)} \quad (eq\ S5)$$

where f_{on} and f_{off} are the fraction of the day the ventilation is on/off (unitless), WS_{on} and WS_{off} is the wind speed (m s^{-1}) when the ventilation is on/off, MW is the molecular weight (g mol^{-1}), T is the room temperature (°C), and c is an empirical sampler constant (unitless). The effective volume, V_{eff}, is then calculated as:

$$V_{\mathrm{eff}} = (V_{\mathrm{PUF}}K_{\mathrm{PUF}})\left[1 - e^{-\left(\frac{R_s}{V_{\mathrm{PUF}}K_{\mathrm{PUF}}}\right)t}\right] \quad (eq\ S6)$$

where V_{PUF} is the PUF volume (m^3) and t is the deployment time (days).

Calculating Concentrations and Emissions

The final concentration in air, C_{air} is:

$$C_{air} = \sum \frac{M_{PCBi}}{V_{eff}} \quad (eq\ S7)$$

where M_{PCBi} is the mass of a PCB (ng).

Emissions, E, are calculated as:

$$E = \sum \frac{M_{PCBi}}{At} \qquad (eq\ S8)$$

where A is the area of the PUF-PES (m^2) and t is the deployment time (days).

Lifetime Cancer Risk Estimation

We estimated the lifetime cancer risk based on both dioxin-like and non-dioxin-like congener contributions. Total risk (TR) is calculated as follows:

$$TR = \frac{C_{PCBi} * IUR * EF * ED * ET * (\frac{1}{24})}{AT} \qquad (eq\ S9)$$

where

C_{PCBi} = measured TEQ (μg/m^3) for dioxin-like congeners; total measured concentration for non-dioxin-like congeners
IUR = inhalation unit risk (μg/m^3)$^{-1}$
EF = exposure frequency (days/year)
ED = exposure duration (years)
ET = exposure time (hours/day)
AT = averaging time, cancer (days)
1/24 = day-hour conversion

To estimate average risk, total risk was calculated for central tendency exposure (CTE) where EF = 170 days/year, ET = 6 hours per day, and ED = 30 years. To estimate a worst-case risk, total risk was calculated for reasonable maximum exposure (RME) where EF = 235 days/year, ET = 9.75 hours per day, and ED = 30 years. To estimate cancer risk from dioxin-like congeners, we used the Inhalation Unit Risk for 2,3,7,8-TCDD[6] and the World Health Organization Toxic Equivalency Factors for dioxin-like PCBs.[7, 8] To estimate cancer risk from non-dioxin like congeners, we used the Inhalation Unit Risk for Aroclor 1254 provided by the EPA.[6] The EPA recommends using the high risk/persistence slope factor when exposures include inhalation of a dust or aerosol contaminated with PCBs or early life exposure for all pathways and PCB mixtures.[9] Values were calculated separately for dioxin- and non-dioxin-like congeners then summed to get a final total risk.

Noncancer Risk Estimation

We estimated the noncancer risk (R_{nc}) from total PCB (sum of dioxin-like and non-dioxin-like congeners) by converting the total air concentration to an oral dose using the following equation.[10] The estimated oral dose was compared to the EPA RfD for Aroclor 1254 to obtain the Hazard Index.[11, 12]

$$R_{nc} = \frac{C_{PCBi} * IR * FTTS}{BW} \qquad (eq\ S10)$$

where

FTTS = (ET x EF) / (24 hours/day x 365 days/year)
C_{PCBi} = total measured concentration for non-dioxin-like congeners (ng m^{-3})

IR = inhalation rate (m^3/day)
FTTS = fraction of total time (over a year) spent indoor at school (unitless)
BW = body weight (kg)
EF = exposure frequency (days/year)
ET = exposure time (hours/day)

Table S4. Summary of school information and deployment details.

School ID	Deployed	Collected	Deployment Time (days)	Lowest Grade	Highest Grade	Total Students (2023)	Year Built or Renovated	Rooms Sampled
SCH A	21-Oct-2023	17-Nov-2023	27	9	12	318	1970	6
SCH B	11-Jul-2023	11-Aug-2023	31	PreK	8	137	1971	11
SCH C	2-Aug-2022	29-Aug-2022	27	PreK	5	247	1959	2
SCH D	4-Aug-2022	23-Aug-2022	19	PreK	12	178	1909†, 1971‡	3
SCH E	23-Jun-2022	25-Jul-2022	32	PreK	6	90	1955	7
SCH F	2-Aug-2022	31-Aug-2022	29	PreK	8	754	1969	2
SCH G	4-Aug-2022	23-Aug-2022	19	PreK	12	362	1937†, 1957‡, 1969‡, 1980‡	3
SCH H	1-Aug-2022	29-Aug-2022	28	PreK	PreK	45*	1974	1
SCH I	20-Jun-2023	21-Jul-2023	31	7	12	339	1969	7
SCH J	2-Aug-2022	29-Aug-2022	27	PreK	5	303	1953	2
SCH K	10-Jul-2023	9-Aug-2023	30	9	12	694	1968	16
SCH L	3-Aug-2022	29-Aug-2022	26	PreK	6	50	1951†, 1988‡	1
SCH M	12-Jun-2022	25-Jul-2022	34	PreK	6	144	1915†, 1954‡	3
SCH N	25-Apr-2023	24-May-2023	29	K	12	77	1960	23
SCH O	23-Apr-2023	22-May-2023	29	PreK	5	213	1970	8
SCH P	4-Aug-2022	23-Aug-2022	19	PreK	12	327	1969†, 1988‡	2

*Most recent enrollment count available (2021)
†Date of original building construction
‡Date of major renovation or addition

Quality Assurance and Quality Control

Table S5. Limit of quantification (LOQ) for each PCB congener or group of co-eluding congeners in units of nanograms. [β]

PCB#	LOQ	PCB#	LOQ	PCB#	LOQ
1	0.0658	46	0.0139	96	0.0091
2	0.0158	48	0.0285	98	0.0066
3	0.0393	49+69	0.1268	99	0.1028
4	0.0765	50+53	0.0640	102	0.0097
5	0.0160	51	0.0150	103	0.0072
6	0.0399	52	0.4881	104	0.0040
7	0.0151	54	0.0060	105	0.0305
8	0.1339	55	0.0071	106	0.0046
9	0.0181	56	0.0216	107	0.0083
10	0.0102	57	0.0062	108+124	0.0130
11	0.1379	58	0.0056	110	0.2002
12+13	0.0137	59+62+75	0.0229	111	0.0040
14	0.0044	60	0.0132	112	0.0039
15	0.0389	61+70 +74+76	0.2537	114	0.0093
16	0.0540	63	0.0085	115	0.0182
17	0.0548	64	0.0742	117	0.0097
18+30	0.1099	66	0.0642	118	0.1113
19	0.0197	67	0.0072	120	0.0039
20+28	0.1178	68	0.0074	121	0.0039
21+33	0.0878	72	0.0054	122	0.0058
22	0.0481	73	0.0048	123	0.0061
23	0.0038	77	0.0034	126	0.0018
24	0.0061	78	0.0040	127	0.0033
25	0.0154	79	0.0041	128+166	0.0138
26+29	0.0295	80	0.0046	129+138 +163	0.1002
27	0.0125	81	0.0045	130	0.0106
31	0.1165	82	0.0208	131	0.0087
32	0.0357	83	0.0201	132	0.0568
34	0.0044	84	0.1083	133	0.0077
35	0.0023	85+116	0.0380	134	0.0144
36	0.0020	86+97+ 109+119	0.0855	135+151	0.0632
37	0.0072	87+125	0.1117	136	0.0502
38	0.0022	88	0.0077	137	0.0175
39	0.0019	89	0.0070	139+140	0.0219
40+71	0.0569	90+101 +113	0.3210	141	0.0238
41	0.0171	91	0.0495	142	0.0061
42	0.0319	92	0.0689	143	0.0071
43	0.0095	93+100	0.0079	144	0.0156
44+47+65	0.2215	94	0.0074	145	0.0075
45	0.0257	95	0.4191	146	0.0165

Table S5 continued

PCB#	LOQ	PCB#	LOQ	PCB#	LOQ
147+149	0.1571	171+173	0.0062	190	0.0151
148	0.0100	172	0.0038	191	0.1339
150	0.0106	174	0.0060	192	0.0181
152	0.0071	175	0.0030	194	0.0102
153+168	0.0945	176	0.0066	195	0.1379
154	0.0145	177	0.0043	196	0.0137
155	0.0077	178	0.0056	197	0.0044
156+157	0.0053	179	0.0136	198+199	0.0389
158	0.0142	180+193	0.0040	200	0.0540
159	0.0027	181	0.0478	201	0.0548
160	0.0050	182	0.1080	202	0.1099
161	0.0081	183	0.0044	203	0.0197
162	0.0030	184	0.0658	205	0.1178
164	0.0073	185	0.0158	206	0.0878
165	0.0051	186	0.0393	207	0.0481
167	0.0030	187	0.0765	208	0.0038
169	0.0021	188	0.0160	209	0.0061
170	0.0050	189	0.0399		

$^{\beta}$The LOQ was calculated as the upper limit of the 99% confidence interval of the log 10 transformed mass in the field blanks (average + 2.325 * standard deviation).

Table S6. Mean surrogate standard recoveries as percentages by congener.

PCB	Mean Recovery (%)	Standard Deviation (%)
13C 3	66	12
13C 15	78	9
13C 31	80	9
13C 52	84	10
13C 118	84	10
13C 153	85	10
13C 180	84	9
13C 194	82	9
13C 206	83	10
13C 209	84	9

Standard Reference Material

We assessed the quality of our method using a standard reference material (NIST, SRM, 2585, Gaithersburg, MD, USA). We added about 0.5 g of SRM to clean PUF (n = 9) and extracted each SRM sample like a typical sample. Our results were compared to the certified concentrations reported by NIST (**Figure S5**). Congeners in parentheses indicate that our analytical method includes other congeners that coelute with the certified congener. Certified results only measure the first listed congener.

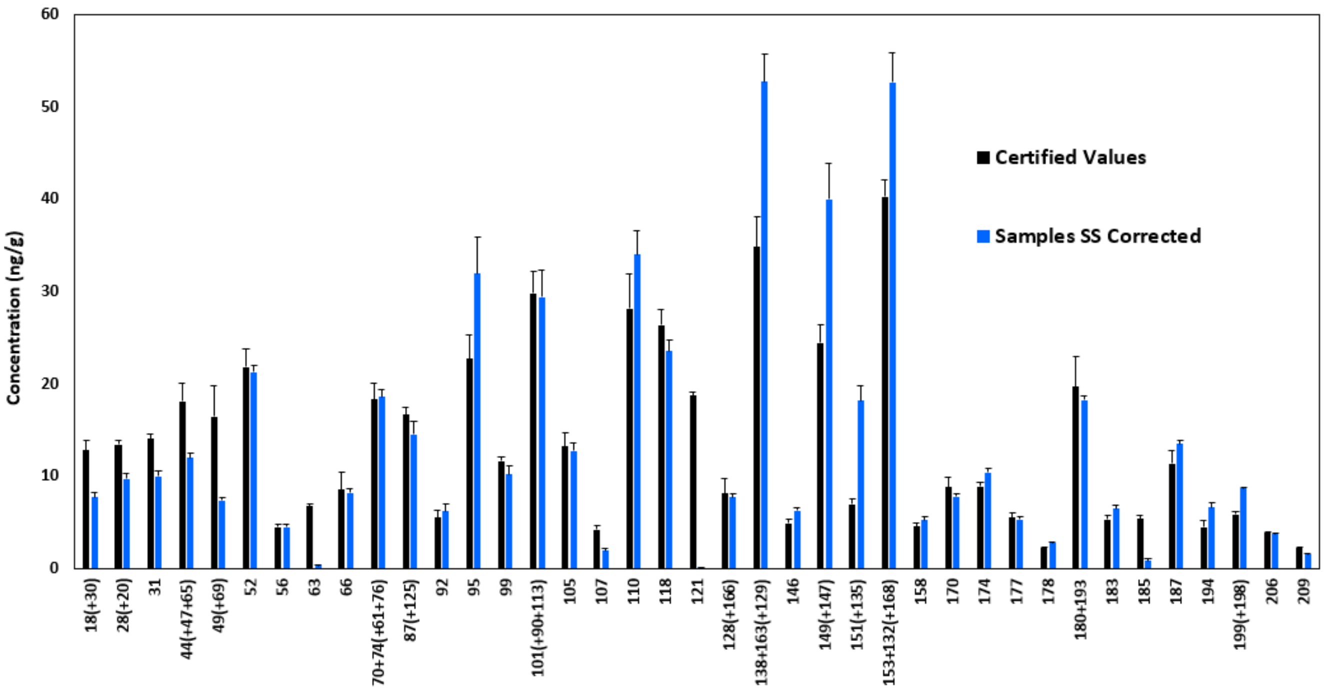

Figure S1. Analysis of National Institutes of Standards and Technology Standard Reference Material (SRM) 2585, organic contaminants in house dust (n = 4).

Negative Controls

Three negative control samples were deployed in SCH N alongside air and emission samples (**Table S8**). A piece of aluminum foil was placed on the ground, over the surface. Emission samplers were then placed over the foil like a normal sample and left in place for 29 days until they were collected with the rest of the samplers. Total mass of PCBs range 10 – 12 ng with an average of 11 ± 1.1 ng. These results are similar to the mass measured in field blanks.

Table S7. Summary of negative control results as mass (ng) and as emissions (ng m^{-2} d^{-1}).

Sample ID	Total Mass	Total Emissions
S466	12	27
S467	12	27
S468	10	22

Results

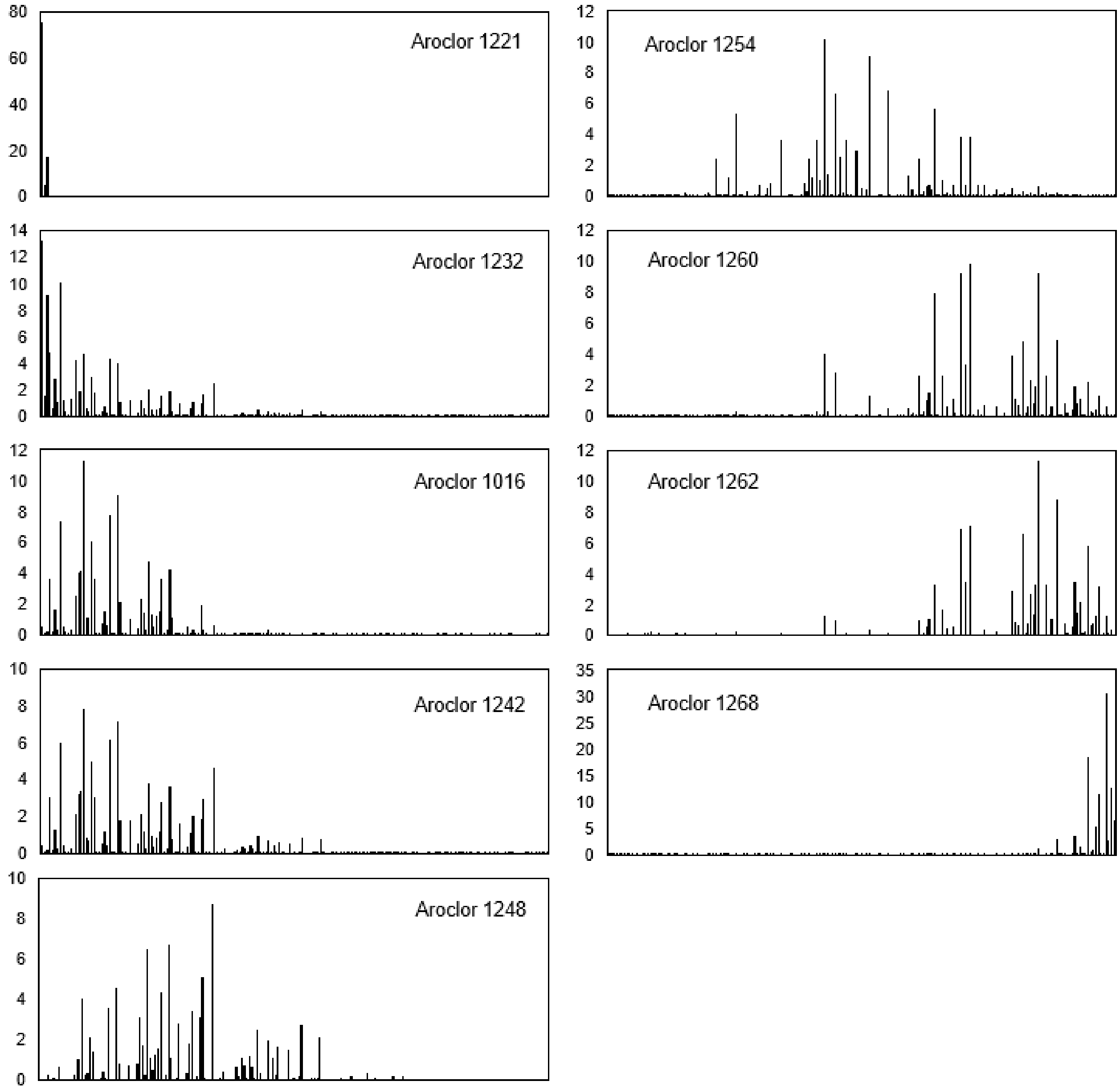

Figure S2. Congener distributions of Aroclors as reported by Rushneck et al.[13] All congeners are shown on the x-axis from 1 to 209. Mass fractions are shown as a percentage on the y-axis.

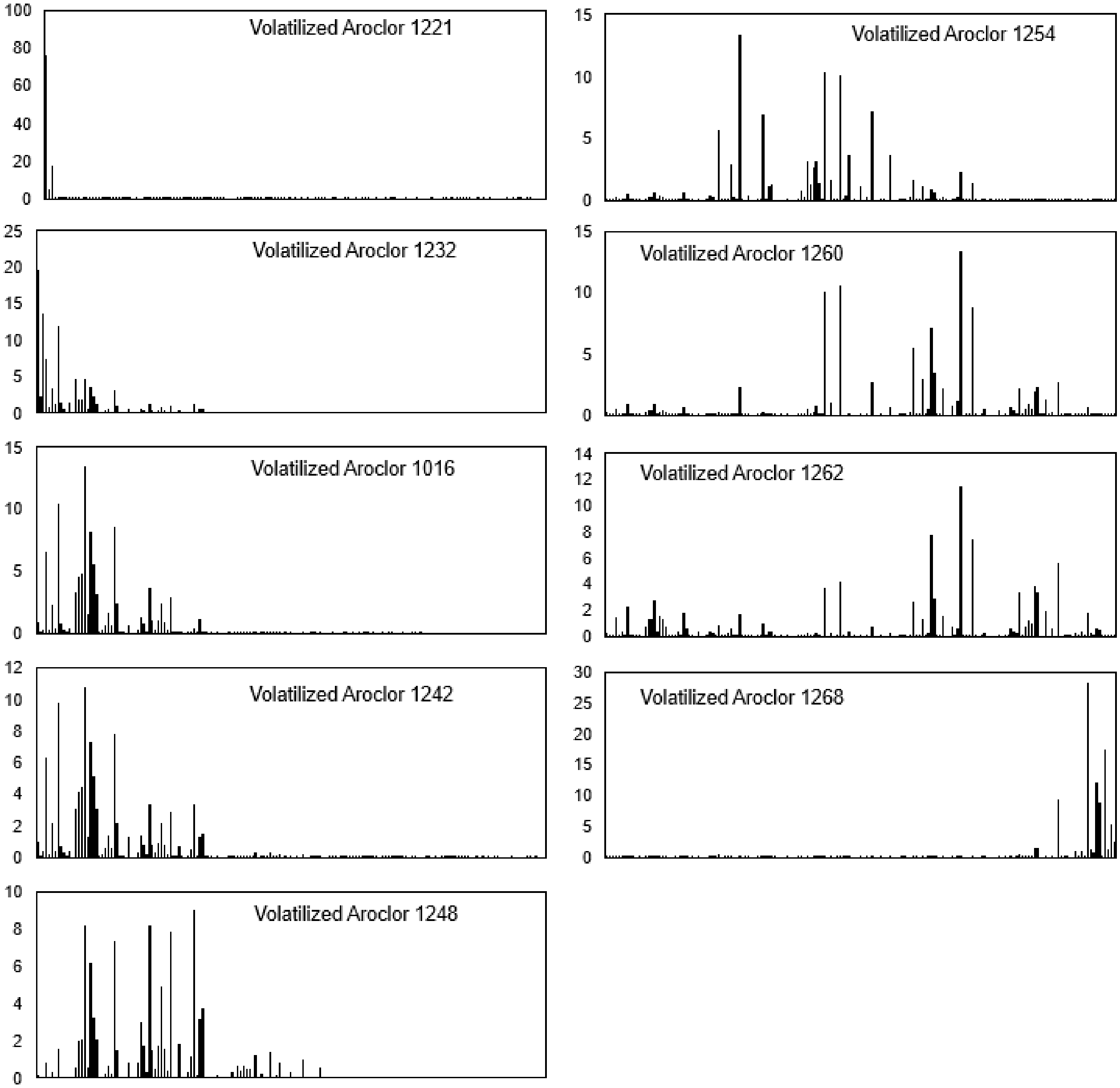

Figure S3. Congener distributions of modeled, volatilized Aroclors using a model described in Hua et al.[14] All congeners are shown on the x-axis from 1 to 209. Mass fractions are shown as a percentage on the y-axis.

Modeled Volatilized Aroclors	Original Aroclors								
	R1221	R1232	R1016	R1242	R1248	R1254	R1260	R1262	R1268
m1221	1.00	0.65	0.03	0.03	0.00	0.00	0.00	0.00	0.00
m1232	0.75	0.97	0.46	0.45	0.20	0.04	0.01	0.01	0.00
m1016	0.04	0.66	0.97	0.89	0.47	0.07	0.01	0.02	0.00
m1242	0.05	0.70	0.96	0.94	0.57	0.13	0.02	0.03	0.00
m1248	0.01	0.48	0.81	0.91	0.93	0.35	0.04	0.03	0.00
m1254	0.01	0.20	0.25	0.40	0.69	0.87	0.27	0.13	0.00
m1260	0.01	0.09	0.09	0.13	0.19	0.68	0.80	0.58	0.03
m1262	0.01	0.20	0.27	0.29	0.24	0.48	0.83	0.75	0.09
m1268	0.00	0.01	0.02	0.02	0.03	0.02	0.20	0.42	0.86

Figure S4. Cos θ values comparing similarities between original Aroclor profiles as reported by Rushneck et al.[13] and modeled volatized Aroclor profiles as described by Hua et al.[14] Values range 0 to 1 where 0 is complete dissimilar and 1 is completely identical.

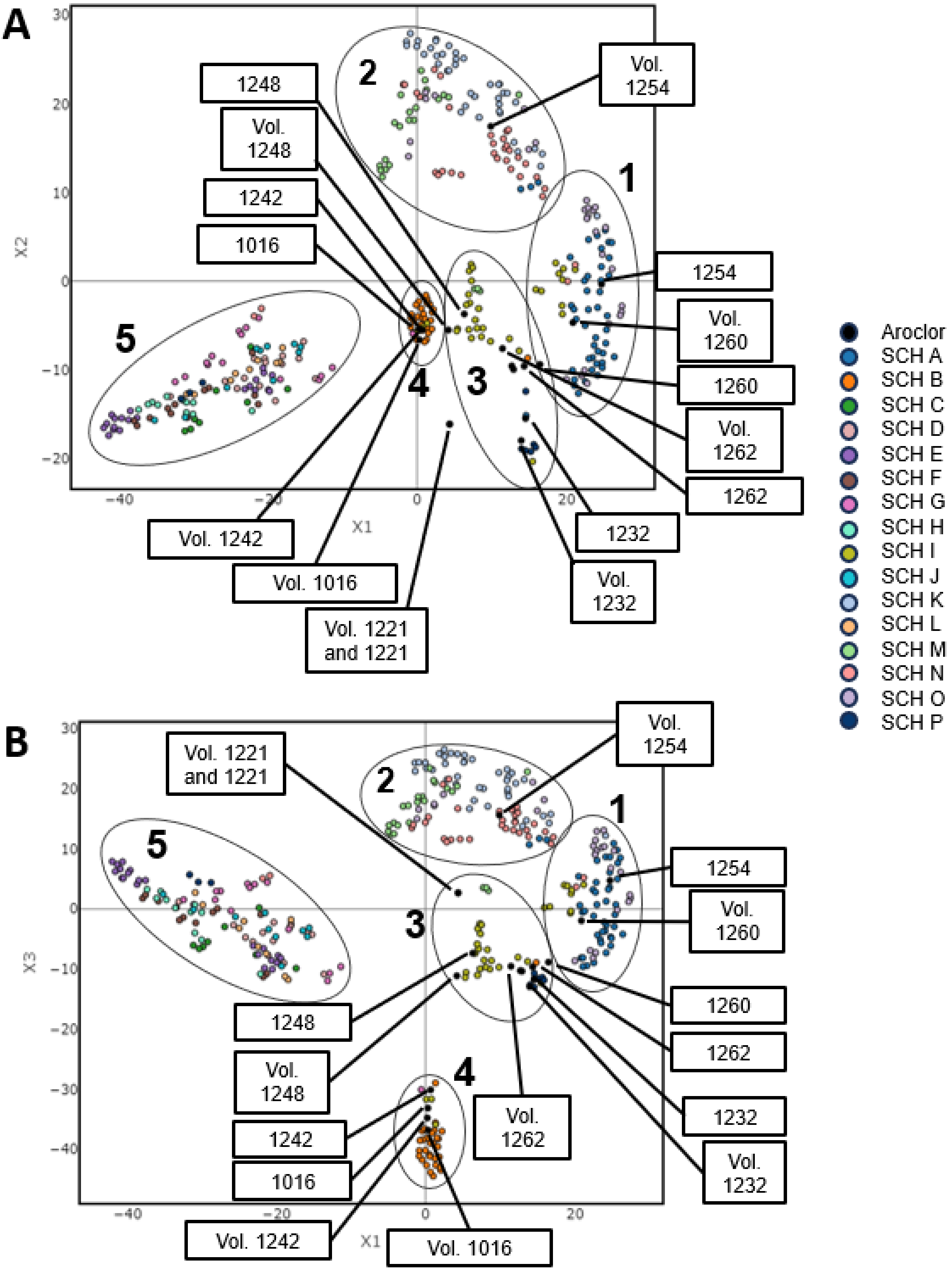

Figure S5. Two perspectives of two-dimensional t-SNE projections with labeled Aroclors (black dots).

Table S8. Summary of all material emissions measured in schools and how different surfaces are categorized in Figure 3.

Material	Category	Count
CMU Wall	CMU Wall	37
Carpet	Carpet	29
Floor Tile	Floor Tile	28
Cove Base	Cove Base	22
Drywall	Drywall	18
Fireproof Coating	Fireproof Coating	9
Roof Fiber Panel	Ceiling	6
Brick Wall	Other Wall	5
Univent Cabinet Top	Other	4
Expansion Joint Sealant	Joint Sealant	4
Mastic	Other	3
Steel Beam	Ceiling	3
Ceiling Tile	Ceiling	2
Storage Cabinet Top	Other	2
Inside Univent	Other	2
Ceiling	Ceiling	1
Painted Fireproof Coating	Fireproof Coating	1
Rubber Floor	Other Flooring	1
Wood Panel	Other Wall	1
Ceiling Joint	Ceiling	1
Glass Blocks	Glass Blocks	1
Gym Mat	Other Wall	1
Gym Floor	Other Flooring	1

Table S9. Comparison of emission ranges (ng m^{-2} d^{-1}) for different materials we hypothesize serve as secondary or tertiary sources in schools with concentrations below the SAL and above the SAL. This list of materials does not include primary sources when known.

	Schools Below SAL			Schools Above SAL		
	Min	Median	Max	Min	Median	Max
CMU Wall	50	87	200	380	4854	45000
Floor Tile	30	58	180	220	2342	28000
Drywall	60	74	430	440	859	16000
Cove Base	50	112	310	260	2640	6100
Carpet	70	124	240	40	359	3900

Table S10. Temperature sensors were deployed and collected with samplers in six schools. Sensors were programmed to record temperatures hourly then averaged by deployment time (~ 30 days).

School	Room	Average Temperature (°C)
SCH A	RM 7	16.7
SCH A	RM 2	20.4
SCH A	RM 8	20.7
SCH A	RM 6	14.7
SCH A	RM 4	15.5
SCH A	RM 3	20.2
SCH A	RM 1	18.1
SCH B	RM 14	21.2
SCH B	RM 11	21.4
SCH B	RM 19	22.9
SCH B	RM 18	24.1
SCH B	RM 17	22.5
SCH I	RM 39	23.1
SCH I	RM 40	23.8
SCH I	RM 41	26.7
SCH I	RM 41	25.7
SCH I	RM 42	26.5
SCH I	RM 42	25.7
SCH I	RM 38	22.0
SCH K	RM 47	23.7
SCH K	RM 48	23.2
SCH K	RM 49	24.8
SCH K	RM 50	24.2
SCH K	RM 51	21.3
SCH K	RM 52	23.8
SCH K	RM 53	21.2
SCH K	RM 55	23.4
SCH K	RM 56	24.7
SCH K	RM 59	26.1
SCH K	RM 59	26.0
SCH K	RM 61	23.2
SCH K	RM 62	25.1
SCH O	RM 93	21.2
SCH N	RM 84	21.6
SCH N	RM 84	25.0
SCH N	RM 72	23.5
SCH N	RM 88	22.5
SCH N	RM 71	23.2
SCH N	RM 77	21.2
SCH N	RM 82	23.0

Table S11. Summary of Aroclors and schools found in each of the five t-SNE clusters.

Cluster	Aroclors	Schools
1	1254, vol. 1260	SCH A, SCH I, SCH K, SCH N, SCH O
2	vol. 1254	SCH A, SCH M, SCH K, SCH N, SCH O
3	1232, vol. 1232, 1248, vol. 1248, 1260, 1262, vol. 1262	SCH B, SCH I, SCH M, SCH P
4	1016, vol. 1016, 1242, vol. 1242	SCH B, SCH G, SCH I
5	non-Aroclor	SCH D, SCH F, SCH C, SCH E, SCH G, SCH H, SCH J, SCH L

Table S12. Estimated lifetime cancer risk calculated with concentration data including both dioxin-like (TEQ) and the sum of all PCB congeners (SUM). Total risk is calculated for central tendency exposure (CTE) and reasonable maximum exposure (RME).

	PCB Concentration (ng m^{-3})		Cancer Risk			
**SID**	**SUM**	**TEQ**	**CTE (EPA) TEQ**	**RME (VT) TEQ**	**CTE (EPA) SUM**	**RME (VT) SUM**
S582	5,500	6.4×10^{-3}	1.2×10^{-5}	2.7×10^{-5}	1.7×10^{-4}	3.8×10^{-4}
S322	3,200	1.2×10^{-2}	2.3×10^{-5}	5.1×10^{-5}	1.1×10^{-4}	2.5×10^{-4}
S436	2,700	3.0×10^{-3}	5.6×10^{-6}	1.3×10^{-5}	8.4×10^{-5}	1.9×10^{-4}
S318	2,700	9.2×10^{-3}	1.7×10^{-5}	3.9×10^{-5}	9.3×10^{-5}	2.1×10^{-4}
S315	2,200	8.4×10^{-3}	1.6×10^{-5}	3.6×10^{-5}	7.8×10^{-5}	1.8×10^{-4}
S583	2,100	2.2×10^{-3}	4.1×10^{-6}	9.3×10^{-6}	6.5×10^{-5}	1.5×10^{-4}
S312	1,600	2.7×10^{-2}	5.1×10^{-5}	1.1×10^{-4}	9.7×10^{-5}	2.2×10^{-4}
S567	1,500	1.3×10^{-3}	2.5×10^{-6}	5.6×10^{-6}	4.6×10^{-5}	1.0×10^{-4}
S321	1,400	3.2×10^{-2}	6.0×10^{-5}	1.4×10^{-4}	1.0×10^{-4}	2.3×10^{-4}
S320	1,300	2.9×10^{-2}	5.4×10^{-5}	1.2×10^{-4}	9.2×10^{-5}	2.1×10^{-4}
S577	990	1.1×10^{-3}	2.1×10^{-6}	4.7×10^{-6}	3.0×10^{-5}	6.8×10^{-5}
S432	750	7.9×10^{-4}	1.5×10^{-6}	3.4×10^{-6}	2.3×10^{-5}	5.1×10^{-5}
S574	700	1.0×10^{-3}	1.9×10^{-6}	4.3×10^{-6}	2.2×10^{-5}	4.9×10^{-5}
S635	630	2.9×10^{-3}	5.5×10^{-6}	1.2×10^{-5}	2.4×10^{-5}	5.3×10^{-5}
S431	580	7.0×10^{-4}	1.3×10^{-6}	3.0×10^{-6}	1.8×10^{-5}	4.0×10^{-5}
S238	570	7.7×10^{-4}	1.5×10^{-6}	3.3×10^{-6}	1.8×10^{-5}	4.0×10^{-5}
S575	570	8.9×10^{-4}	1.7×10^{-6}	3.8×10^{-6}	1.8×10^{-5}	4.0×10^{-5}
S523	550	1.5×10^{-3}	2.9×10^{-6}	6.4×10^{-6}	1.9×10^{-5}	4.2×10^{-5}
S281	540	8.8×10^{-4}	1.7×10^{-6}	3.8×10^{-6}	1.7×10^{-5}	3.8×10^{-5}
S368	520	1.2×10^{-3}	2.3×10^{-6}	5.2×10^{-6}	1.7×10^{-5}	3.9×10^{-5}
S370	410	6.0×10^{-4}	1.1×10^{-6}	2.5×10^{-6}	1.3×10^{-5}	2.9×10^{-5}
S435	400	3.4×10^{-4}	6.5×10^{-7}	1.5×10^{-6}	1.2×10^{-5}	2.7×10^{-5}
S437	350	3.4×10^{-4}	6.4×10^{-7}	1.4×10^{-6}	1.1×10^{-5}	2.4×10^{-5}
S634	300	1.0×10^{-3}	1.9×10^{-6}	4.4×10^{-6}	1.1×10^{-5}	2.4×10^{-5}
S576	290	2.7×10^{-4}	5.2×10^{-7}	1.2×10^{-6}	8.7×10^{-6}	1.9×10^{-5}
S400	290	3.3×10^{-4}	6.2×10^{-7}	1.4×10^{-6}	8.9×10^{-6}	2.0×10^{-5}
S565	280	2.6×10^{-4}	4.9×10^{-7}	1.1×10^{-6}	8.4×10^{-6}	1.9×10^{-5}
S427	250	2.7×10^{-4}	5.2×10^{-7}	1.2×10^{-6}	7.7×10^{-6}	1.7×10^{-5}
S566	240	3.4×10^{-4}	6.4×10^{-7}	1.4×10^{-6}	7.3×10^{-6}	1.7×10^{-5}
S383	240	6.6×10^{-4}	1.3×10^{-6}	2.8×10^{-6}	8.2×10^{-6}	1.8×10^{-5}
S663	240	1.2×10^{-3}	2.3×10^{-6}	5.2×10^{-6}	9.2×10^{-6}	2.1×10^{-5}
S664	240	1.2×10^{-3}	2.3×10^{-6}	5.1×10^{-6}	9.2×10^{-6}	2.1×10^{-5}
S360	230	3.4×10^{-4}	6.5×10^{-7}	1.5×10^{-6}	7.1×10^{-6}	1.6×10^{-5}
S405	220	2.3×10^{-4}	4.3×10^{-7}	9.7×10^{-7}	6.6×10^{-6}	1.5×10^{-5}

Table S12 continued

S289	190	1.9×10^{-4}	3.7×10^{-7}	8.2×10^{-7}	5.7×10^{-6}	1.3×10^{-5}
S361	190	2.5×10^{-4}	4.8×10^{-7}	1.1×10^{-6}	5.9×10^{-6}	1.3×10^{-5}
S366	190	7.7×10^{-4}	1.5×10^{-6}	3.3×10^{-6}	7.0×10^{-6}	1.6×10^{-5}
S570	180	1.5×10^{-4}	2.8×10^{-7}	6.2×10^{-7}	5.4×10^{-6}	1.2×10^{-5}
S428	170	1.8×10^{-4}	3.5×10^{-7}	7.8×10^{-7}	5.1×10^{-6}	1.1×10^{-5}
S424	170	1.8×10^{-4}	3.4×10^{-7}	7.6×10^{-7}	5.1×10^{-6}	1.2×10^{-5}
S377	170	1.6×10^{-4}	3.0×10^{-7}	6.8×10^{-7}	5.2×10^{-6}	1.2×10^{-5}
S072	170	6.6×10^{-4}	1.2×10^{-6}	2.8×10^{-6}	6.2×10^{-6}	1.4×10^{-5}
S283	160	2.4×10^{-4}	4.5×10^{-7}	1.0×10^{-6}	4.9×10^{-6}	1.1×10^{-5}
S426	160	1.8×10^{-4}	3.4×10^{-7}	7.6×10^{-7}	5.0×10^{-6}	1.1×10^{-5}
S018	150	8.6×10^{-5}	1.6×10^{-7}	3.7×10^{-7}	4.3×10^{-6}	9.7×10^{-6}
S422	150	1.7×10^{-4}	3.3×10^{-7}	7.4×10^{-7}	4.5×10^{-6}	1.0×10^{-5}
S397	150	2.0×10^{-4}	3.8×10^{-7}	8.4×10^{-7}	4.6×10^{-6}	1.0×10^{-5}
S161	150	8.8×10^{-5}	1.7×10^{-7}	3.8×10^{-7}	4.5×10^{-6}	1.0×10^{-5}
S430	150	2.3×10^{-4}	4.3×10^{-7}	9.7×10^{-7}	4.8×10^{-6}	1.1×10^{-5}
S621	150	4.5×10^{-4}	8.6×10^{-7}	1.9×10^{-6}	5.1×10^{-6}	1.1×10^{-5}
S016	140	7.9×10^{-5}	1.5×10^{-7}	3.4×10^{-7}	4.1×10^{-6}	9.2×10^{-6}
S423	140	1.1×10^{-4}	2.1×10^{-7}	4.8×10^{-7}	4.2×10^{-6}	9.5×10^{-6}
S649	140	3.2×10^{-4}	6.1×10^{-7}	1.4×10^{-6}	4.5×10^{-6}	1.0×10^{-5}
S662	140	7.0×10^{-4}	1.3×10^{-6}	3.0×10^{-6}	5.3×10^{-6}	1.2×10^{-5}
S069	140	6.3×10^{-4}	1.2×10^{-6}	2.7×10^{-6}	5.3×10^{-6}	1.2×10^{-5}
S406	130	1.6×10^{-4}	3.1×10^{-7}	7.0×10^{-7}	4.0×10^{-6}	9.0×10^{-6}
S607	120	5.0×10^{-5}	9.5×10^{-8}	2.1×10^{-7}	3.5×10^{-6}	7.8×10^{-6}
S564	120	9.4×10^{-5}	1.8×10^{-7}	4.0×10^{-7}	3.6×10^{-6}	8.2×10^{-6}
S545	120	3.6×10^{-5}	6.9×10^{-8}	1.5×10^{-7}	3.6×10^{-6}	8.1×10^{-6}
S418	110	1.3×10^{-4}	2.5×10^{-7}	5.7×10^{-7}	3.3×10^{-6}	7.4×10^{-6}
S419	110	1.7×10^{-4}	3.3×10^{-7}	7.3×10^{-7}	3.6×10^{-6}	8.1×10^{-6}
S648	110	2.7×10^{-4}	5.1×10^{-7}	1.2×10^{-6}	3.8×10^{-6}	8.5×10^{-6}
S624	110	3.8×10^{-4}	7.2×10^{-7}	1.6×10^{-6}	3.9×10^{-6}	8.7×10^{-6}
S626	110	4.1×10^{-4}	7.7×10^{-7}	1.7×10^{-6}	4.0×10^{-6}	9.0×10^{-6}
S544	99	1.7×10^{-5}	3.2×10^{-8}	7.3×10^{-8}	2.9×10^{-6}	6.4×10^{-6}
S606	96	5.5×10^{-5}	1.0×10^{-7}	2.3×10^{-7}	2.8×10^{-6}	6.4×10^{-6}
S569	94	1.3×10^{-4}	2.5×10^{-7}	5.6×10^{-7}	2.9×10^{-6}	6.6×10^{-6}
S317	93	1.8×10^{-4}	3.3×10^{-7}	7.5×10^{-7}	3.0×10^{-6}	6.7×10^{-6}
S070	93	4.4×10^{-4}	8.3×10^{-7}	1.9×10^{-6}	3.5×10^{-6}	7.8×10^{-6}
S425	91	8.8×10^{-5}	1.7×10^{-7}	3.7×10^{-7}	2.8×10^{-6}	6.2×10^{-6}
S602	90	3.3×10^{-5}	6.3×10^{-8}	1.4×10^{-7}	2.6×10^{-6}	5.9×10^{-6}
S404	85	8.4×10^{-5}	1.6×10^{-7}	3.6×10^{-7}	2.6×10^{-6}	5.8×10^{-6}
S608	79	3.6×10^{-5}	6.9×10^{-8}	1.5×10^{-7}	2.3×10^{-6}	5.2×10^{-6}
S612	77	3.9×10^{-4}	7.3×10^{-7}	1.7×10^{-6}	2.9×10^{-6}	6.6×10^{-6}
S600	74	5.4×10^{-5}	1.0×10^{-7}	2.3×10^{-7}	2.2×10^{-6}	5.0×10^{-6}
S316	74	1.5×10^{-4}	2.8×10^{-7}	6.2×10^{-7}	2.4×10^{-6}	5.4×10^{-6}

Table S12 continued

S571	73	5.2×10^{-5}	9.9×10^{-8}	2.2×10^{-7}	2.2×10^{-6}	4.9×10^{-6}
S314	63	1.2×10^{-4}	2.3×10^{-7}	5.3×10^{-7}	2.0×10^{-6}	4.6×10^{-6}
S609	59	1.4×10^{-5}	2.6×10^{-8}	5.8×10^{-8}	1.7×10^{-6}	3.8×10^{-6}
S568	59	8.8×10^{-5}	1.7×10^{-7}	3.8×10^{-7}	1.8×10^{-6}	4.1×10^{-6}
S572	52	3.6×10^{-5}	6.8×10^{-8}	1.5×10^{-7}	1.5×10^{-6}	3.5×10^{-6}
S065	50	5.1×10^{-5}	9.7×10^{-8}	2.2×10^{-7}	1.5×10^{-6}	3.4×10^{-6}
S605	50	4.1×10^{-5}	7.8×10^{-8}	1.8×10^{-7}	1.5×10^{-6}	3.4×10^{-6}
S650	49	1.1×10^{-4}	2.0×10^{-7}	4.6×10^{-7}	1.6×10^{-6}	3.6×10^{-6}
S549	47	1.7×10^{-5}	3.2×10^{-8}	7.2×10^{-8}	1.4×10^{-6}	3.1×10^{-6}
S657	44	1.6×10^{-4}	3.0×10^{-7}	6.8×10^{-7}	1.6×10^{-6}	3.5×10^{-6}
S654	43	1.4×10^{-4}	2.6×10^{-7}	5.9×10^{-7}	1.5×10^{-6}	3.3×10^{-6}
S396	42	4.9×10^{-5}	9.3×10^{-8}	2.1×10^{-7}	1.3×10^{-6}	2.9×10^{-6}
S655	42	1.5×10^{-4}	2.8×10^{-7}	6.4×10^{-7}	1.5×10^{-6}	3.3×10^{-6}
S083	40	6.2×10^{-5}	1.2×10^{-7}	2.7×10^{-7}	1.3×10^{-6}	2.8×10^{-6}
S643	39	1.9×10^{-4}	3.6×10^{-7}	8.2×10^{-7}	1.5×10^{-6}	3.3×10^{-6}
S063b	37	3.4×10^{-5}	6.5×10^{-8}	1.5×10^{-7}	1.1×10^{-6}	2.5×10^{-6}
S620	36	1.6×10^{-4}	3.0×10^{-7}	6.7×10^{-7}	1.3×10^{-6}	3.0×10^{-6}
S656	31	1.1×10^{-4}	2.0×10^{-7}	4.5×10^{-7}	1.1×10^{-6}	2.4×10^{-6}
S616	29	5.3×10^{-5}	1.0×10^{-7}	2.3×10^{-7}	9.1×10^{-7}	2.1×10^{-6}
S543	27	1.3×10^{-5}	2.4×10^{-8}	5.4×10^{-8}	8.0×10^{-7}	1.8×10^{-6}
S313	26	5.2×10^{-5}	9.8×10^{-8}	2.2×10^{-7}	8.5×10^{-7}	1.9×10^{-6}
S417	26	7.4×10^{-5}	1.4×10^{-7}	3.2×10^{-7}	8.7×10^{-7}	2.0×10^{-6}
S548	25	6.9×10^{-5}	1.3×10^{-7}	2.9×10^{-7}	8.4×10^{-7}	1.9×10^{-6}
S573	24	2.7×10^{-5}	5.2×10^{-8}	1.2×10^{-7}	7.4×10^{-7}	1.7×10^{-6}
S603	23	1.2×10^{-5}	2.2×10^{-8}	5.0×10^{-8}	6.7×10^{-7}	1.5×10^{-6}
S550	23	2.5×10^{-5}	4.8×10^{-8}	1.1×10^{-7}	6.9×10^{-7}	1.5×10^{-6}
S604	22	1.1×10^{-5}	2.0×10^{-8}	4.5×10^{-8}	6.5×10^{-7}	1.5×10^{-6}
S551	19	7.2×10^{-6}	1.4×10^{-8}	3.1×10^{-8}	5.7×10^{-7}	1.3×10^{-6}
S498	18	1.4×10^{-5}	2.7×10^{-8}	6.0×10^{-8}	5.3×10^{-7}	1.2×10^{-6}
S420	18	9.6×10^{-6}	1.8×10^{-8}	4.1×10^{-8}	5.4×10^{-7}	1.2×10^{-6}
S601	16	7.5×10^{-6}	1.4×10^{-8}	3.2×10^{-8}	4.8×10^{-7}	1.1×10^{-6}
S584	16	2.3×10^{-5}	4.5×10^{-8}	1.0×10^{-7}	4.9×10^{-7}	1.1×10^{-6}
S403	15	1.5×10^{-5}	2.9×10^{-8}	6.5×10^{-8}	4.5×10^{-7}	1.0×10^{-6}
S399	13	1.2×10^{-5}	2.4×10^{-8}	5.3×10^{-8}	3.8×10^{-7}	8.6×10^{-7}
S121	13	5.5×10^{-6}	1.0×10^{-8}	2.4×10^{-8}	3.9×10^{-7}	8.9×10^{-7}
S421	11	8.8×10^{-6}	1.7×10^{-8}	3.7×10^{-8}	3.2×10^{-7}	7.1×10^{-7}
S401	11	9.8×10^{-6}	1.9×10^{-8}	4.2×10^{-8}	3.3×10^{-7}	7.5×10^{-7}
S499	8.7	1.5×10^{-5}	2.8×10^{-8}	6.2×10^{-8}	2.8×10^{-7}	6.2×10^{-7}
S500	8.1	9.7×10^{-6}	1.8×10^{-8}	4.1×10^{-8}	2.5×10^{-7}	5.6×10^{-7}
S035	7.5	8.1×10^{-6}	1.5×10^{-8}	3.5×10^{-8}	2.3×10^{-7}	5.1×10^{-7}
S259	6.8	2.3×10^{-5}	4.3×10^{-8}	9.8×10^{-8}	2.4×10^{-7}	5.4×10^{-7}
S501	5.9	1.4×10^{-5}	2.6×10^{-8}	5.9×10^{-8}	2.0×10^{-7}	4.4×10^{-7}

**Table S12 continued**						
S402	5.8	8.6×10^{-6}	1.6×10^{-8}	3.7×10^{-8}	1.8×10^{-7}	4.1×10^{-7}
S192	5.7	7.6×10^{-6}	1.4×10^{-8}	3.3×10^{-8}	1.8×10^{-7}	3.9×10^{-7}
S041	5.4	4.4×10^{-6}	8.4×10^{-9}	1.9×10^{-8}	1.6×10^{-7}	3.6×10^{-7}
S193	5.4	5.4×10^{-6}	1.0×10^{-8}	2.3×10^{-8}	1.6×10^{-7}	3.7×10^{-7}
S153	5.1	4.6×10^{-6}	8.7×10^{-9}	2.0×10^{-8}	1.5×10^{-7}	3.4×10^{-7}
S038	4.9	6.3×10^{-6}	1.2×10^{-8}	2.7×10^{-8}	1.5×10^{-7}	3.4×10^{-7}
S398	4.8	5.3×10^{-6}	1.0×10^{-8}	2.3×10^{-8}	1.5×10^{-7}	3.3×10^{-7}
S152	4.8	7.6×10^{-6}	1.4×10^{-8}	3.2×10^{-8}	1.5×10^{-7}	3.4×10^{-7}
S221	4.8	9.6×10^{-6}	1.8×10^{-8}	4.1×10^{-8}	1.5×10^{-7}	3.5×10^{-7}
S044	4.7	4.6×10^{-6}	8.7×10^{-9}	1.9×10^{-8}	1.4×10^{-7}	3.2×10^{-7}
S151	4.5	6.4×10^{-6}	1.2×10^{-8}	2.7×10^{-8}	1.4×10^{-7}	3.1×10^{-7}
S008	4.4	6.4×10^{-6}	1.2×10^{-8}	2.7×10^{-8}	1.4×10^{-7}	3.1×10^{-7}
S032	4.1	4.9×10^{-6}	9.3×10^{-9}	2.1×10^{-8}	1.3×10^{-7}	2.9×10^{-7}
S033	4.0	2.5×10^{-6}	4.8×10^{-9}	1.1×10^{-8}	1.2×10^{-7}	2.6×10^{-7}
S042	4.0	3.7×10^{-6}	7.0×10^{-9}	1.6×10^{-8}	1.2×10^{-7}	2.7×10^{-7}
S039	3.9	2.5×10^{-6}	4.8×10^{-9}	1.1×10^{-8}	1.1×10^{-7}	2.6×10^{-7}
S036	3.9	2.8×10^{-6}	5.4×10^{-9}	1.2×10^{-8}	1.2×10^{-7}	2.6×10^{-7}
S019	3.8	5.6×10^{-6}	1.1×10^{-8}	2.4×10^{-8}	1.2×10^{-7}	2.7×10^{-7}
S015	3.6	5.0×10^{-6}	9.5×10^{-9}	2.1×10^{-8}	1.1×10^{-7}	2.5×10^{-7}
S043	3.5	3.5×10^{-6}	6.6×10^{-9}	1.5×10^{-8}	1.0×10^{-7}	2.4×10^{-7}
S004	3.3	3.1×10^{-6}	5.9×10^{-9}	1.3×10^{-8}	1.0×10^{-7}	2.2×10^{-7}
S165	3.3	3.2×10^{-6}	6.1×10^{-9}	1.4×10^{-8}	1.0×10^{-7}	2.3×10^{-7}
S009	3.1	3.4×10^{-6}	6.4×10^{-9}	1.4×10^{-8}	9.4×10^{-8}	2.1×10^{-7}
S014	3.0	3.1×10^{-6}	5.8×10^{-9}	1.3×10^{-8}	9.3×10^{-8}	2.1×10^{-7}
S186	3.0	9.4×10^{-6}	1.8×10^{-8}	4.0×10^{-8}	1.0×10^{-7}	2.3×10^{-7}
S063	2.9	4.2×10^{-6}	8.0×10^{-9}	1.8×10^{-8}	9.1×10^{-8}	2.1×10^{-7}
S011	2.9	3.9×10^{-6}	7.4×10^{-9}	1.7×10^{-8}	9.1×10^{-8}	2.0×10^{-7}
S080	2.8	2.6×10^{-6}	5.0×10^{-9}	1.1×10^{-8}	8.4×10^{-8}	1.9×10^{-7}
S022	2.8	2.5×10^{-6}	4.8×10^{-9}	1.1×10^{-8}	8.5×10^{-8}	1.9×10^{-7}
S005	2.8	5.6×10^{-6}	1.1×10^{-8}	2.4×10^{-8}	9.1×10^{-8}	2.0×10^{-7}
S176	2.6	6.3×10^{-6}	1.2×10^{-8}	2.7×10^{-8}	8.6×10^{-8}	1.9×10^{-7}
S208	2.6	1.7×10^{-5}	3.3×10^{-8}	7.3×10^{-8}	1.1×10^{-7}	2.4×10^{-7}
S013	2.5	2.9×10^{-6}	5.5×10^{-9}	1.2×10^{-8}	7.8×10^{-8}	1.7×10^{-7}
S081	2.4	2.1×10^{-6}	3.9×10^{-9}	8.8×10^{-9}	7.1×10^{-8}	1.6×10^{-7}
S173	2.4	3.2×10^{-6}	6.1×10^{-9}	1.4×10^{-8}	7.5×10^{-8}	1.7×10^{-7}
S020	2.2	2.3×10^{-6}	4.4×10^{-9}	1.0×10^{-8}	6.6×10^{-8}	1.5×10^{-7}
S185	2.1	8.4×10^{-6}	1.6×10^{-8}	3.6×10^{-8}	7.5×10^{-8}	1.7×10^{-7}
S231	2.0	1.3×10^{-5}	2.5×10^{-8}	5.5×10^{-8}	8.1×10^{-8}	1.8×10^{-7}
S256	1.8	3.5×10^{-6}	6.6×10^{-9}	1.5×10^{-8}	5.7×10^{-8}	1.3×10^{-7}
S210	1.8	9.0×10^{-6}	1.7×10^{-8}	3.8×10^{-8}	6.9×10^{-8}	1.5×10^{-7}
S209	1.7	1.1×10^{-5}	2.2×10^{-8}	4.8×10^{-8}	6.9×10^{-8}	1.6×10^{-7}
S007	0.4	2.6×10^{-6}	5.0×10^{-9}	1.1×10^{-8}	1.6×10^{-8}	3.6×10^{-8}

Table S12 continued						
S012	0.3	2.5×10^{-6}	4.7×10^{-9}	1.1×10^{-8}	1.3×10^{-8}	3.0×10^{-8}
S006	0.3	2.2×10^{-6}	4.2×10^{-9}	9.3×10^{-9}	1.3×10^{-8}	2.8×10^{-8}

Table S13. Mean and median air concentrations (ng m^{-3}) grouped by how often rooms are occupied by students.

Room Type	Min	Mean	Median	Standard Deviation	Max	n
Constant Occupancy						
Classroom	2	230	30	690	5,650	100
Intermittent Use						
Gymnasium	120	310	260	250	620	5
Library	48	460	600	360	730	3
Auditorium	42	NA	NA	NA	42	1
Locker Room	33	43	46	7	48	4
Hallway	67	110	89	61	200	4
Minimal Student Access						
Office	5	100	90	100	310	13
Utility Room	11	720	110	1,110	3,560	22

Table S14. Mean field blank masses compared to mean sample masses by school. The total mass of PCBs from field blanks are insignificant compared to the total mass in the samples.

School	Field Blank Mass Mean (ng)	Number of Field Blanks	Sample Mass Mean (ng)	Number of Samples	Blank Fraction
A	13	7	24,000	45	0.1%
B	14	4	1,200	30	1.2%
C	1	3	100	14	1.3%
D	2	2	70	12	2.6%
E	3	2	120	8	2.3%
F	2	3	50	21	4.2%
G	3	3	60	15	4.6%
H	5	2	70	11	8.2%
I	12	5	5,600	32	0.2%
J	8	2	30	10	23.4%
K	25	5	26,000	40	0.1%
L	7	3	40	9	17.6%
M	6	3	1,600	23	0.4%
N	6	5	2,900	34	0.2%
O	13	3	19,000	27	0.1%
P	6	1	1,200	9	0.5%

Table S15. Comparison of PCB concentrations from Vermont schools and previous studies in homes and apartments.

Study	Sampler	Min	Median	Mean	Max	Method
This Study	PAS	1.7	59	281	5700	All congeners
Harrad 2006[15]	PAS	0.5	1.8	2.8	9.8	Six congeners times 5[a]
Menichini 2007[16]	low-vol.	9.5	NA	NA	41.5	Six congeners times 5[a]
Wilson 2011[17]	low-vol.	0.3	7.9	12.8	114.3	Eighty four congeners
Zhang 2011[18]	PAS	0.8	NA	NA	130.5	Seventy two congeners
Frederiksen 2012[19]	low-vol.	168	859	1030	3843	Six congeners times 5[a]
Audy 2018[20]	PAS	0.1	0.455	0.734	5.1	Seven congeners times 5[b]
Audy 2018[20]	PAS	0.139	0.467	0.661	4.23	Seven congeners times 5[b]
Herkert 2018[21]	PAS	0.45	NA	2.83	6.97	All congeners
Andersen 2020[22]	low-vol.	NA	2164	2330	NA	Seven congeners times 5[b]
Andersen 2021[23]	low-vol.	58	NA	NA	948	Seven congeners times 5[b]

[a] ∑PCB = 5 x (∑congeners 28, 52, 101, 138, 153, 180)
[b] ∑PCB = 5 x (∑congeners 28, 52, 101, 118, 138, 153, 180)

References

(1) Hua, J. B. X.; Marek, R. F.; Jones, M. P.; Erb, T.; Owen, S. C.; Hornbuckle, K. C. *Dataset for PCBs in school air: wide-spread emissions of airborne PCBs from building materials in 98 school rooms in Vermont* (Dataset) Iowa Research Online. 2024. DOI: 10.25820/data.007328.

(2) *Method 1668C Chlorinated Biphenyl Congeners in Water, Soil, Sediment, Biosolids, and Tissue by HRGC/HRMS;* 2010. https://www.epa.gov/sites/default/files/2015-09/documents/method_1668c_2010.pdf

(3) Herkert, N. J.; Hornbuckle, K. C. Effects of room airflow on accurate determination of PUF-PAS sampling rates in the indoor environment. *Environmental Science: Processes & Impacts* **2018**, *20* (5), 757-766, 10.1039/C8EM00082D. DOI: 10.1039/C8EM00082D.

(4) Shoeib, M.; Harner, T. Characterization and Comparison of Three Passive Air Samplers for Persistent Organic Pollutants. *Environmental Science & Technology* **2002**, *36* (19), 4142-4151. DOI: 10.1021/es020635t.

(5) Herkert, N. J.; Martinez, A.; Hornbuckle, K. C. A Model Using Local Weather Data to Determine the Effective Sampling Volume for PCB Congeners Collected on Passive Air Samplers. *Environmental Science & Technology* **2016**, *50* (13), 6690-6697. DOI: 10.1021/acs.est.6b00319.

(6) EPA, U. *Regional Screening Levels (RSLs) - Generic Tables*. US EPA, 2024. https://www.epa.gov/risk/regional-screening-levels-rsls-generic-tables (accessed.

(7) EPA, U. *Documents for Recommended Toxicity Equivalency Factors for Human Health Risk Assessments of Dioxin and Dioxin-Like Compounds*. US EPA, 2024. https://www.epa.gov/risk/documents-recommended-toxicity-equivalency-factors-human-health-risk-assessments-dioxin-and (accessed.

(8) Van den Berg, M.; Birnbaum, L.; Bosveld, A. T.; Brunström, B.; Cook, P.; Feeley, M.; Giesy, J. P.; Hanberg, A.; Hasegawa, R.; Kennedy, S. W.; et al. Toxic equivalency factors (TEFs) for PCBs, PCDDs, PCDFs for humans and wildlife. *Environmental health perspectives* **1998**, *106* (12), 775-792. DOI: 10.1289/ehp.98106775 PubMed.

(9) EPA, U. *Regional Screening Levels (RSLs) - Frequent Questions*. US EPA, 2024. https://www.epa.gov/risk/regional-screening-levels-rsls-frequent-questions#FQ50 (accessed.

(10) US Environmental Protection Agency: *PCB Exposure Estimation Tool*; 2.1. 2020. Obtained from EPA Region 1 PCB Coordinator, not available online. (accessed September 2021).

(11) *Risk Assessment Guidance for Superfund: Volume I - Human Health Evaluation Manual (Part A);* 1989. https://www.epa.gov/risk/risk-assessment-guidance-superfund-rags-part

(12) *Aroclor 1254 CASRN 11097-69-1;* U.S. Environmental Protection Agency, 1994. https://iris.epa.gov/ChemicalLanding/&substance_nmbr=389

(13) Rushneck, D. R.; Beliveau, A.; Fowler, B.; Hamilton, C.; Hoover, D.; Kaye, K.; Berg, M.; Smith, T.; Telliard, W. A.; Roman, H.; et al. Concentrations of dioxin-like PCB congeners in unweathered Aroclors by HRGC/HRMS using EPA Method 1668A. *Chemosphere* **2004**, *54* (1), 79-87. DOI: https://doi.org/10.1016/S0045-6535(03)00664-7.

(14) Hua, J. B. X.; Marek, R. F.; Hornbuckle, K. C. Polyurethane Foam Emission Samplers to Identify Sources of Airborne Polychlorinated Biphenyls from Glass-Block Windows and Other Room Surfaces in a Vermont School. *Environmental Science & Technology* **2023**, *57* (38), 14310-14318. DOI: 10.1021/acs.est.3c05195.

(15) Harrad, S.; Hazrati, S.; Ibarra, C. Concentrations of Polychlorinated Biphenyls in Indoor Air and Polybrominated Diphenyl Ethers in Indoor Air and Dust in Birmingham, United Kingdom: Implications for Human Exposure. *Environmental Science & Technology* **2006**, *40* (15), 4633-4638. DOI: 10.1021/es0609147.

(16) Menichini, E.; Iacovella, N.; Monfredini, F.; Turrio-Baldassarri, L. Relationships between indoor and outdoor air pollution by carcinogenic PAHs and PCBs. *Atmospheric Environment* **2007**, *41* (40), 9518-9529. DOI: https://doi.org/10.1016/j.atmosenv.2007.08.041.
(17) Wilson, L. R.; Palmer, P. M.; Belanger, E. E.; Cayo, M. R.; Durocher, L. A.; Hwang, S.-A. A.; Fitzgerald, E. F. Indoor Air Polychlorinated Biphenyl Concentrations in Three Communities Along the Upper Hudson River, New York. *Archives of Environmental Contamination and Toxicology* **2011**, *61* (3), 530-538. DOI: 10.1007/s00244-010-9627-x.
(18) Zhang, X.; Diamond, M. L.; Robson, M.; Harrad, S. Sources, Emissions, and Fate of Polybrominated Diphenyl Ethers and Polychlorinated Biphenyls Indoors in Toronto, Canada. *Environmental Science & Technology* **2011**, *45* (8), 3268-3274. DOI: 10.1021/es102767g.
(19) Frederiksen, M.; Meyer, H. W.; Ebbehøj, N. E.; Gunnarsen, L. Polychlorinated biphenyls (PCBs) in indoor air originating from sealants in contaminated and uncontaminated apartments within the same housing estate. *Chemosphere* **2012**, *89* (4), 473-479. DOI: https://doi.org/10.1016/j.chemosphere.2012.05.103.
(20) Audy, O.; Melymuk, L.; Venier, M.; Vojta, S.; Becanova, J.; Romanak, K.; Vykoukalova, M.; Prokes, R.; Kukucka, P.; Diamond, M. L.; et al. PCBs and organochlorine pesticides in indoor environments - A comparison of indoor contamination in Canada and Czech Republic. *Chemosphere* **2018**, *206*, 622-631. DOI: 10.1016/j.chemosphere.2018.05.016 From NLM.
(21) Herkert, N. J.; Jahnke, J. C.; Hornbuckle, K. C. Emissions of Tetrachlorobiphenyls (PCBs 47, 51, and 68) from Polymer Resin on Kitchen Cabinets as a Non-Aroclor Source to Residential Air. *Environmental Science & Technology* **2018**, *52* (9), 5154-5160. DOI: 10.1021/acs.est.8b00966.
(22) Andersen, H. V.; Gunnarsen, L.; Knudsen, L. E.; Frederiksen, M. PCB in air, dust and surface wipes in 73 Danish homes. *International Journal of Hygiene and Environmental Health* **2020**, *229*, 113429. DOI: https://doi.org/10.1016/j.ijheh.2019.113429.
(23) Andersen, H. V.; Kolarik, B.; Nielsen, N. S.; Hougaard, T.; Gunnarsen, L.; Knudsen, L. E.; Frederiksen, M. Indoor air concentrations of PCB in a contaminated building estate and factors of importance for the variance. *Building and Environment* **2021**, *204*, 108135. DOI: https://doi.org/10.1016/j.buildenv.2021.108135.
